# Disease Biomarker Detection Using Fluorescence Polarization Assays: Principles and Clinical Applications

**DOI:** 10.3390/diagnostics16183050

**Published:** 2026-09-20

**Authors:** Liliya I. Mukhametova, Sergei A. Eremin, Andreas K. Tsakalof

**Affiliations:** 1 Faculty of Chemistry, M.V. Lomonosov Moscow State University, Leninsky Gory 1/3, 119991 Moscow, Russia; eremin_sergei@hotmail.com or eremin@enz.chem.msu.ru; 2 Department of Medicine, University of Thessaly, Biopolis, 41500 Larissa, Greece

**Keywords:** fluorescence polarization, biomarkers, homogeneous immunoassay, aptasensor, infection diagnostics, cancer diagnostics, therapeutic drug monitoring, point-of-care

## Abstract

Modern clinical diagnostics require highly sensitive, rapid, and technologically simple methods for quantitatively determining biomarkers directly at the patient’s bedside. Fluorescence polarization (FP) meets these criteria, as it enables the homogeneous recording of an analytical signal based on changes in the rotational diffusion of a fluorescently labeled ligand upon binding to a target, without separation or washing steps. This comprehensive narrative review analyzes the literature over the past 10–15 years devoted to the use of FP analysis for detecting biomarkers of socially significant diseases. The fundamental principles of the method and modern analysis formats are discussed—from classical fluorescence polarization immunoassay (FPIA) with antibodies to aptamer sensors, DNAzyme-based systems, and reagent-free biosensors such as Quenchbody. Particular attention is paid to new approaches to signal amplification, including those using nanoparticles, protein aggregation, and isothermal amplification of nucleic acids. The main part of the review is devoted to practical applications of FP: diagnostics of infectious diseases (brucellosis, tuberculosis, and viral infections) demonstrating sensitivity at the level of traditional ELISA; liquid biopsy in oncology, including the detection of extracellular vesicles using aptamer FP platforms; therapeutic drug monitoring of antibiotics in whole blood on portable paper media; and the identification of biomarkers for metabolic and ophthalmological disorders. Prospects for integrating polarization detection with CRISPR/Cas systems, microfluidics, and smartphone-compatible readers are discussed, paving the way to the creation of a new generation of inexpensive devices for personalized medicine.

## 1. Introduction

Biomarkers are fundamental to modern biomedicine, serving as objective indicators for assessing physiological and pathological processes, as well as responses to therapeutic interventions. According to the MarkerDB definition, “a biomarker is a measurable substance or characteristic of an organism that provides evidence of some phenomenon, such as disease, diet, or environmental exposure” [1]. This definition encompasses a broad spectrum of analytes—from classic metabolites (glucose, cholesterol) and proteins (tumor markers, cardiac troponins) to nucleic acids (mutations, microRNAs) and even physical parameters [2,3,4,5,6]. Despite significant progress, the accurate and rapid quantification of biomarkers in complex biological matrices (blood, urine, saliva, cerebrospinal fluid) remains challenging, primarily due to low concentrations of many markers at early disease stages, interfering matrix components, and the need for high specificity and reproducibility in miniaturized, high-throughput formats.

In response to these challenges, methods based on fluorescence polarization (FP) and anisotropy (FA) have attracted considerable interest. The principal advantage of FP-based assays over heterogeneous immunoassays such as ELISA and many CLIA formats is that FP assays are homogeneous (“mix-and-measure”) assays that do not require separation, immobilization, or washing steps [7,8]. This substantially reduces assay time, labor intensity, and the risk of procedural errors, while facilitating automation, high-throughput analysis, and decreased turnaround time (or results waiting time) a critical criterion for clinical utility and large-scale population screening. Moreover, FP assays can be implemented on compact or even smartphone-based devices and are thus well suited to point-of-care (POC), decentralized biomarker screening, enabling rapid detection in community-based or out-of-hospital settings [9,10].

**Figure 1:**
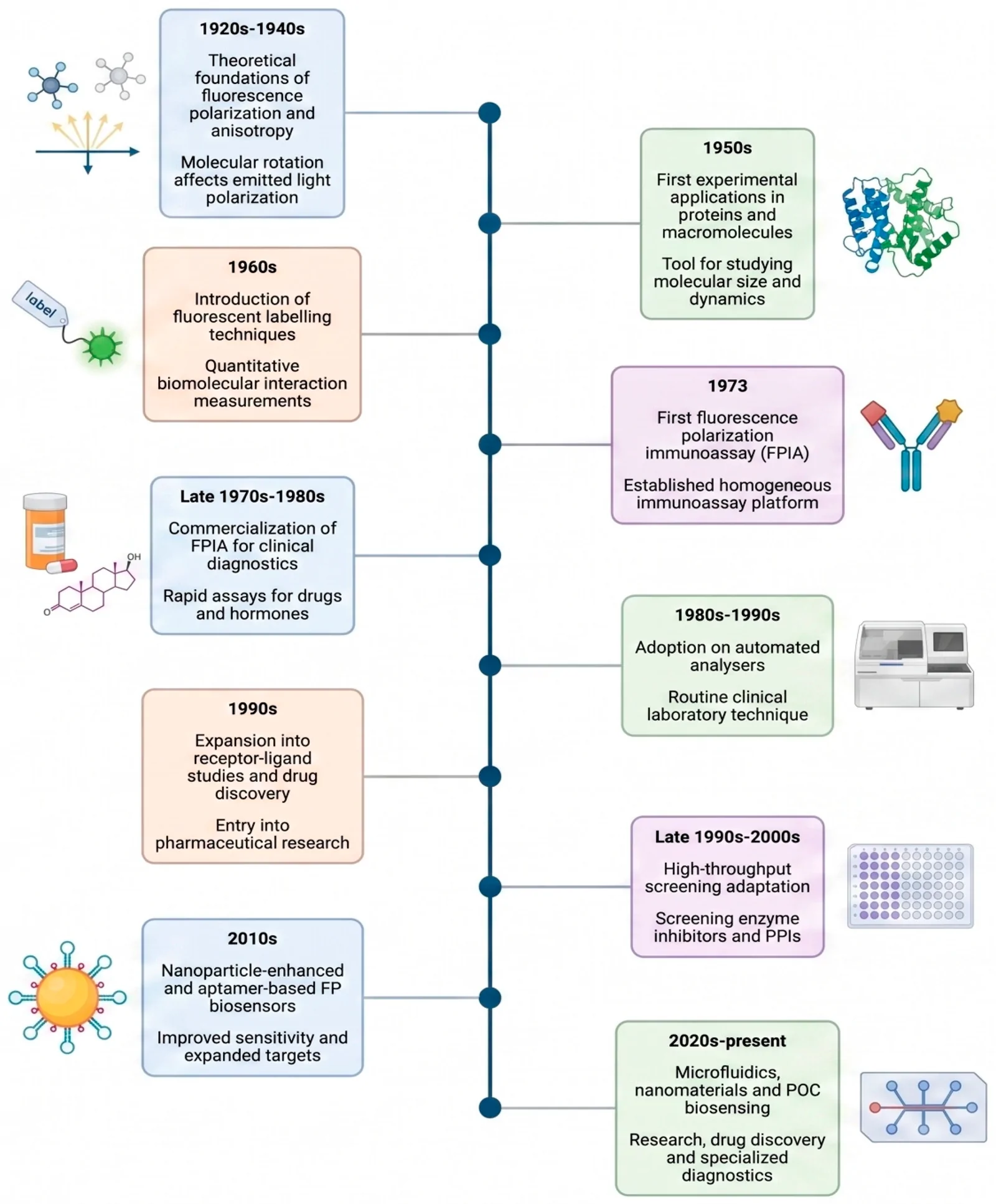
Historical timeline of FP assays development and clinical applications.

The development of FP assays has progressed from first-generation competitive immunoassays for small-molecule analysis in the 1970s, through molecular interaction and high-throughput screening formats in the 1990s–2000s, to modern amplification-assisted, nanoparticle-enhanced, aptamer-based, and microfluidic biosensing platforms that substantially expand the analytical scope and sensitivity of FP measurements [11,12,13,14] (Figure 1). However, despite this technological evolution, the clinical translation of FP methods remains limited compared to established heterogeneous techniques.

Several comprehensive reviews have addressed FP principles and selected applications [7,8,15,16,17]. Nevertheless, a critical analysis that compares FP performance across different biomarker classes—small molecules, proteins, nucleic acids, and whole viral particles—and evaluates the real-world effectiveness of emerging signal amplification strategies and novel recognition elements is still lacking. Furthermore, the specific technical barriers that prevent widespread FP adoption in routine clinical diagnostics and POC settings have not been examined.

The aim of this review is to summarize and critically analyse the current state of the art in the development and application of fluorescence polarization/anisotropy (FP/FA)-based sensors employing various recognition elements for the quantitative detection of biomarkers of diverse chemical nature, including nucleic acids, antibodies, proteins, and small molecules. This review aims to help researchers and developers of clinical analysis systems select optimal configurations of FP/FA sensors for specific biomarker targets, as well as to stimulate the implementation of these rapid, homogeneous and highly sensitive methods in practical laboratory diagnostics.

For this narrative review, we performed a targeted literature search in PubMed, Scopus, Web of Science, and Google Scholar covering the period from January 2010 to March 2026 (with emphasis on the last 10–15 years, as stated in the Abstract). The final search date was 15 May 2026. The search strategy employed combinations of the following keywords and their synonyms: fluorescence polarization, fluorescence anisotropy, FPIA, homogeneous immunoassay, aptamer-based FP, nucleic acid FP, CRISPR-assisted FP, biomarkers, point-of-care, therapeutic drug monitoring, infection, cancer diagnostics. Boolean operators (AND, OR) were adapted to the syntax of each database.

Inclusion criteria: (1) original research or review articles in English; (2) development or application of fluorescence polarization/anisotropy-based methods for detection of human biomarkers; (3) presence of experimental data (in buffer, biological fluids, or clinical samples). Exclusion criteria: (1) studies performed exclusively in animals without demonstrated relevance to human biomarkers; (2) studies not related to human disease diagnostics; (3) articles where FP was used solely as a physicochemical technique without analytical or clinical context. As this is a narrative review, we did not aim to exhaustively cover all publications, nor did we perform formal risk-of-bias assessment according of review guidelines Using this search strategy, 110 publications were identified and analyzed in this review.

This review addresses three main objectives: (1) to summarize and compare the analytical performance—including limits of detection (LOD), specificity, dynamic range, and turnaround time—of FP-based assays for key high-molecular-weight biomarkers of infectious, oncological, metabolic, and autoimmune diseases, classified by analyte molecular weight; (2) to critically evaluate the effectiveness of various signal amplification strategies (nanoparticles, enzymatic amplification, isothermal nucleic acid amplification) and the use of non-traditional recognition elements (aptamers, nanobodies, affibodies) in overcoming the classical limitation of FP—the small change in molecular volume when detecting large analytes—and to identify which approaches have proven most successful; (3) to identify the main technical barriers (matrix effects, temperature sensitivity, insufficient portability, lack of standardization) that still hinder the widespread implementation of FP in point-of-care diagnostics and to discuss how emerging technologies—including microfluidics, paper-based devices, smartphone-compatible readers, and CRISPR/Cas systems—are addressing these challenges. By answering these questions, this review aims to provide researchers and developers with evidence-based guidance for selecting optimal FP assay configurations for specific biomarker targets and to stimulate further translation of these rapid, homogeneous, and sensitive methods into practical laboratory and clinical applications.

## 2. Fluorescence Polarization (FP): Principle and Assay Formats

The fundamental principle of fluorescence polarization (FP) is that the polarization of emitted fluorescence depends on the rotational mobility of the fluorophore during its excited-state lifetime (typically 1–100 ns) [15]. When a fluorescent molecule is excited by plane-polarized light with a wavelength corresponding to the absorption maximum of the fluorophore, it absorbs photons preferentially if its dipole moment is oriented parallel to the polarization vector of the exciting light. Between photon absorption and fluorescence emission, the molecule may rotate in space [15,18]. The extent of rotation depends on two factors: the fluorescence lifetime of the fluorophore (a characteristic constant for each label) and the rotational diffusion rate, which is inversely proportional to the hydrodynamic volume of the molecule and the viscosity of the medium.

The relationship between observed polarization and molecular properties is described by the Perrin equation:

(1) $$\frac{1}{P}-\frac{1}{3}=\left(\frac{1}{P_{0}}-\frac{1}{3}\right)\left(1+\frac{RT}{\eta V}\tau \right)$$

where P is the observed polarization, R is the universal gas constant, T is the absolute temperature, η is the viscosity of the solution, P_0_ is the internal polarization, V is the hydrodynamic volume of fluorophore, and τ is the excited state fluorescence lifetime.

At constant temperature and viscosity, Equation (1) shows that the observed FP is directly dependent on the hydrodynamic volume of the fluorescent species [14]. Consequently, FP can monitor biological processes that involve changes in molecular size—such as binding interactions, enzymatic cleavage, or conformational changes—provided the resulting volume change is within the detectable range of the technique.

**Figure 2:**
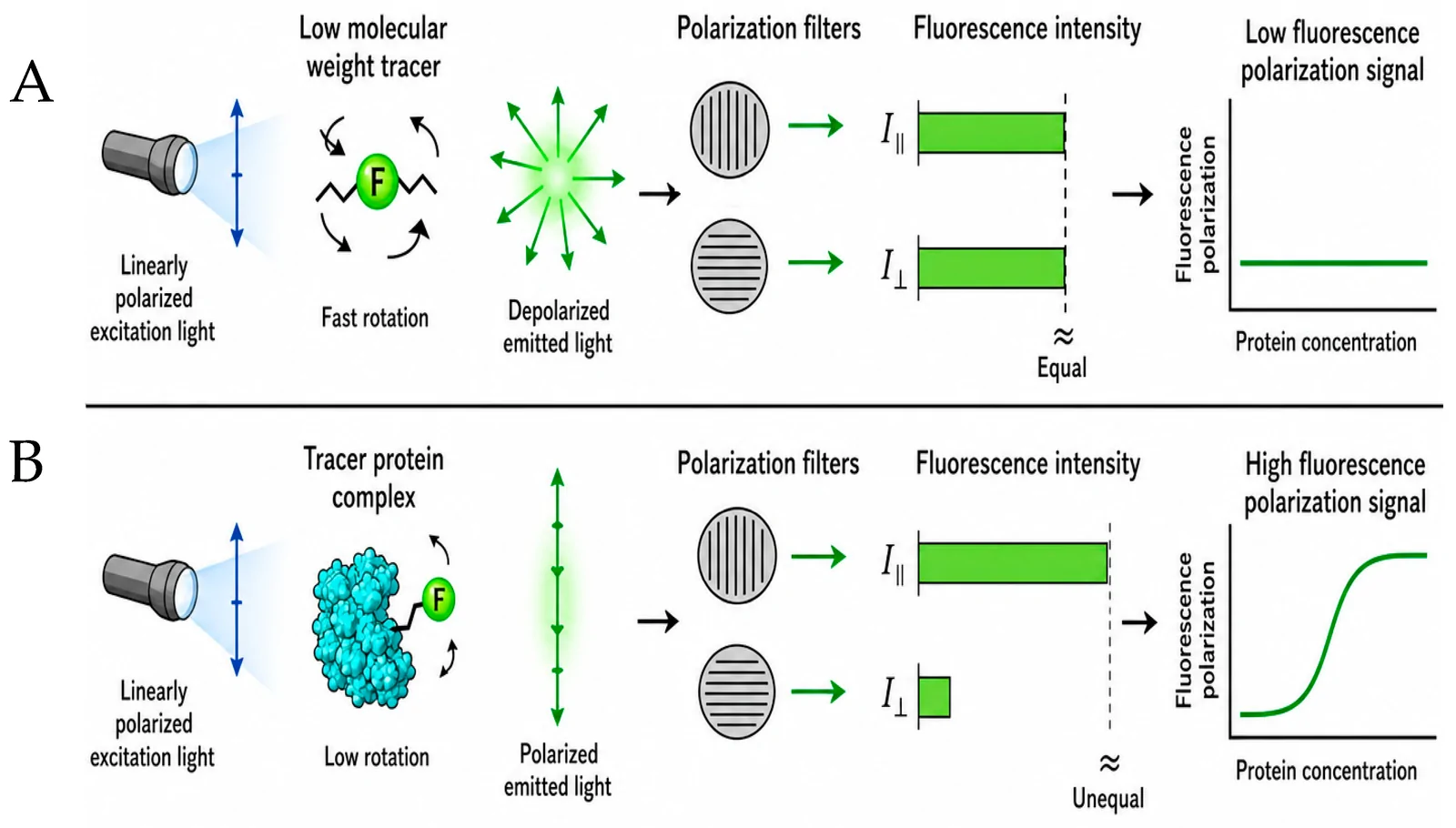
Principles of the fluorescence polarization assay (FPA). The degree of polarization depends on the rotation rate of the fluorescent compound/complex: (**A**) low molecular weight fluorescent molecule rotates rapidly and the emitted fluorescence is depolarized, (**B**) larger complexes rotate more slowly, resulting in reduced depolarization and increased fluorescence polarization.

When a small fluorescent molecule is excited by plane-polarized light, it rotates rapidly during the excited-state lifetime, undergoing multiple reorientations before emitting a photon (Figure 2A). As a result, the emitted fluorescence is depolarized, meaning the intensities of the vertically and horizontally polarized components are approximately equal. Conversely, when the same fluorophore is covalently attached to a larger molecule (or binds to a macromolecular target), its rotational diffusion is significantly reduced. The molecule undergoes minimal rotation during the excited state, and the emitted fluorescence retains a high degree of polarization (Figure 2B).

The measured signal in the fluorescence polarization (FP) method is quantitatively expressed by two closely related, but not identical, quantities: polarization (P) and anisotropy (A). Both are determined by the intensities of the vertically (I∥) and horizontally (I⊥) polarized components of the radiation excited by vertically polarized light [7]. It is more convenient to use millipolarization (mFP), where mFP = FP × 1000 (Equation (2)). To correct for instrumental bias (i.e., unequal detection efficiency of the two polarization channels), a G-factor is introduced. The G-factor is determined experimentally, typically using a free fluorophore solution with known anisotropy or by using horizontally polarized excitation. The corrected anisotropy (FA) and polarization (FP) are then calculated as:

(2) $$mFP=\frac{I_{∥}-GI_{⊥}}{I_{∥}+GI_{⊥}}\times 1000;\;mFA=\frac{I_{∥}-GI_{⊥}}{I_{∥}+2GI_{⊥}}\times 1000$$

FP-fluorescence polarization, FA-fluorescence anisotropy, G-factor calibration is essential for obtaining instrument-independent values and for reliable comparison of data across different platforms.

The sensitivity of any FP assay is governed by the change in hydrodynamic volume (or molecular size) upon binding, which affects the rotational correlation time. For a spherical particle, the rotational correlation time (ϕ) is given by the Stokes–Einstein–Debye relation:

(3) $$\phi =\frac{\eta \cdot V_{h}}{k_{B}\cdot T}$$

where η is the solvent viscosity, V_h_ is the hydrodynamic volume, k_B_ is the Boltzmann constant, and T is the absolute temperature. For non-spherical molecules, shape factors must be included, but the above expression provides a useful approximation.

The hardware of an FP instrument imposes practical limits on sensitivity, reproducibility, and accuracy. Every component—from the polarizer to the detector—contributes to the final signal quality. The polarizer determines the polarization purity of the excitation light. Film polarizers are inexpensive but have low transmission in the UV; wire-grid polarizers are wavelength-dependent; prism polarizers offer the highest purity but are bulky and costly. For routine clinical work in the visible range, film polarizers are often chosen, while prism polarizers are reserved for research at extreme wavelengths.

The detector is a key element in determining the signal-to-noise ratio. Photomultiplier tubes (PMTs) offer the highest sensitivity for weak signals. Avalanche photodiodes (APDs) provide a good compromise between sensitivity, dynamic range, and robustness. Silicon photodiodes are used in low-cost, portable devices where extreme sensitivity is not required. The ultimate physical detection limit, however, is dictated by photon statistics and the quantum efficiency of the detector, as well as by the fluorophore’s brightness and photostability.

The choice of platform defines the application scope—from benchtop analyzers to field-portable devices. The Abbott TDx analyzer (introduced in 1981) became a gold standard for automated homogeneous immunoassays of small molecules (e.g., drugs and hormones), eliminating labor-intensive separation steps and providing excellent reproducibility (CV 4–7%) and high correlation with reference methods (r = 0.97–0.99) [19,20]. Modern technology now enables pocket-sized FP analyzers capable of performing up to 500 measurements with sample volumes as low as 1 µL. Recently, smartphone-based detectors with 3D-printed housings (weighing 175 g, costing ~$300) have been reported, which interface with the phone camera for rapid analysis [21]. Ultimately, all hardware specifications are not just numbers—they define the practical analytical capabilities. The proper selection of polarizers and detectors, along with careful G-factor calibration, allows the user to match the instrument to the specific analytical task and to reliably assess the quality of the obtained data.

**Figure 3:**
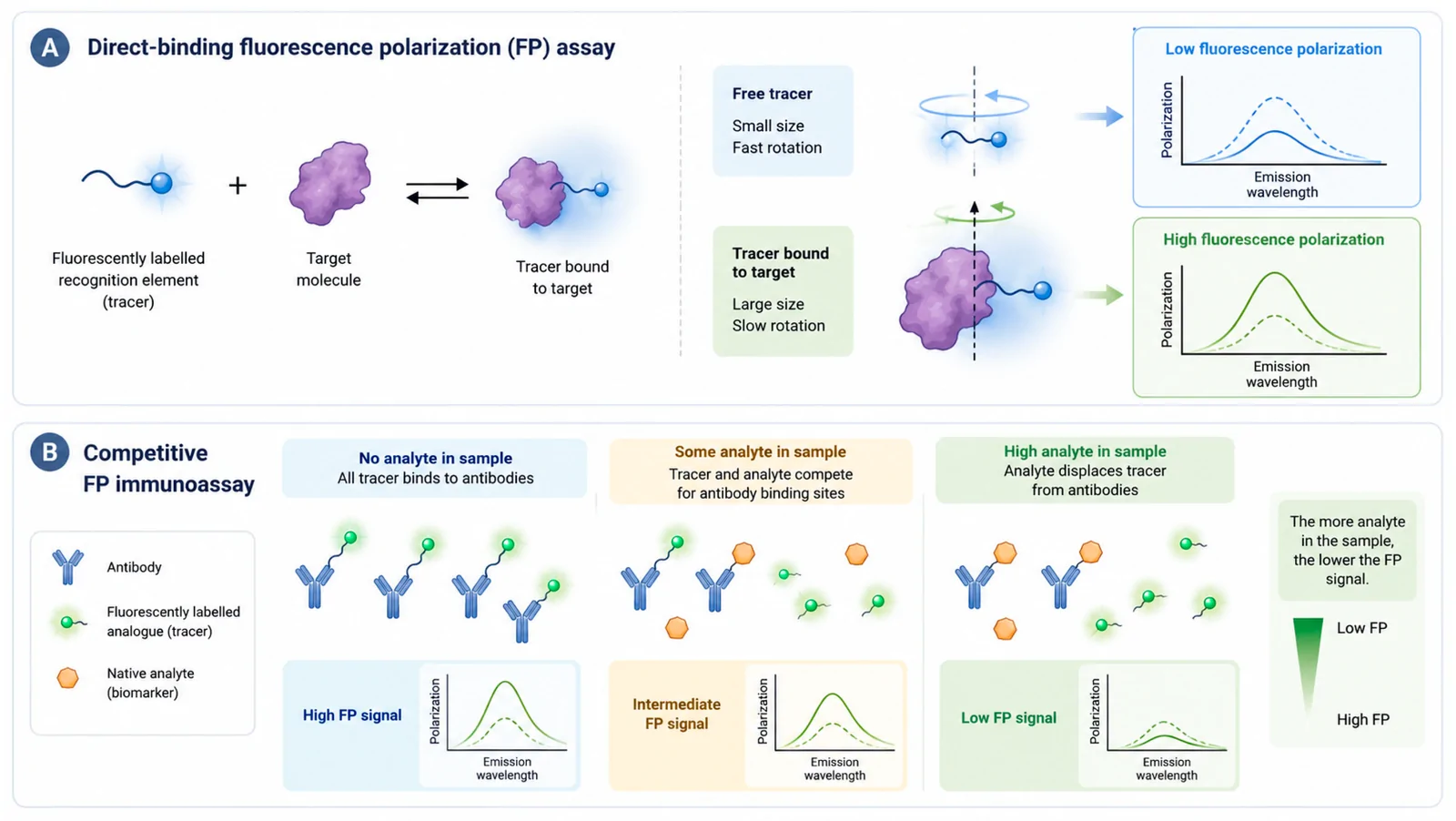
Direct-binding (**A**) and competitive (**B**) assay formats. Direct binding is particularly well siuted for high-molecular-weight targets, while competitive immunoassay is optimal for low-molecular-weight compounds.

FP is a versatile analytical technique with several assay formats that can be selected depending on the properties of the analyte. In the direct-binding fluorescence polarization (FP) assay format (Figure 2 and Figure 3A), a fluorescently labelled recognition element (tracer) binds to a target molecule. The resulting change in FP signal depends on the difference in rotational correlation times between the free and bound states. Consequently, the increase in polarization is greatest when the tracer is much smaller than the target molecule. This format is particularly well suited for the quantification of high-molecular-weight targets, such as peptides, proteins (e.g., receptors, enzymes, and DNA-binding proteins), and other macromolecules.

For low-molecular-weight analytes (e.g., a hormone, drug, toxin, or small peptide) the competitive FP immunoassay is applied, when target analyte and fluorescently labelled analogue (tracer) compete for a limited number of antibody binding sites (Figure 3B). If the biomarker is absent from the sample, all the tracer binds to the antibodies, resulting in a high FP signal. If the biomarker is abundant in the sample, it displaces the tracer from the antibody complex. The tracer remains free in solution, and the FP signal decreases (Figure 3B).

Tracer synthesis is a key step in producing highly specific immunoreagents. A tracer consists of three components: an antigen (or hapten), a fluorophore, and a linker between them. The choice of each tracer component is critical for the sensitivity and specificity of the assay.

Historically, fluorescein has been the most commonly used fluorophore in classical FPIAs due to its unique properties—high quantum yield (~0.9), the availability of reactive derivatives (e.g., FITC for binding to amino groups), and compatibility with most commercial FP analyzers originally designed for this wavelength [7,22]. However, the range of dyes currently available has expanded significantly, and the choice of fluorophore is determined primarily by the type of biological matrix in which the assay is performed and the required sensitivity (Table 1).

For study of complex biological fluids (serum, plasma, whole blood), dyes with excitation and emission wavelengths > 600 nm (e.g., HiLyte Fluor 647, Alexa Fluor 647, Cy5, ATTO 647N) are preferred, since they minimize the contribution of autofluorescence of endogenous chromophores (bilirubin, porphyrins, NADH) [26]. For buffer systems or diluted samples, green dyes (FITC, Alexa Fluor 488, TAMRA) can be used, which are inexpensive and produce a high signal. In ultrasensitive assays and when working with highly fluorescent matrices, the use of lanthanide chelates (Eu^3+^, Tb^3+^) with fluorescence lifetimes in the microsecond range, allowing for time-resolved detection, is promising [32]. Quantum dots or dye sets with non-overlapping spectra (e.g., FITC + TAMRA + Cy5) are suitable for multiplex analysis [33]. A comparative description of the main fluorophores used in FP assays is presented in Table 1.

An equally important element of the tracer is the linker (spacer), which connects the fluorophore and the recognition moiety. Its length and chemical nature influence: fluorescence quenching: short linkers can lead to contact of the fluorophore with electron-rich regions of the protein or nucleic acid; binding affinity: an incorrectly selected linker can change the conformation of the hapten and reduce the binding constant to the antibody; and rotational mobility: long hydrophilic linkers (e.g., PEG_3_–PEG_12_) provide greater freedom of rotation, which can improve the dynamic range of the FP signal [31]. The influence of linker length has been discussed in detail in previous reviews [7,13,22]. Thus, optimal tracer design requires a coordinated choice of all three components, considering the nature of the analyte, the type of sample, and the instrumentation used.

FPA is not the only competitive fluorescence polarization immunoassay. The competitive FP principle is used in many assay types. The difference is in binding partner for analyte and the fluorescent tracer (Table 2).

## 3. Application of FP Assays for Human Disease Diagnosis by Monitoring Disease Biomarkers

Biomarkers play a key role in modern disease diagnostics, prognosis, and monitoring. There are numerous ways to classify biomarkers, from their chemical nature to their clinical application (diagnostic, prognostic, predictive) [34]. However, when developing analytical methods, particularly those using fluorescence polarization, the most fundamental classification of biomarkers is by molecular weight. The FPIA method is effectively used to detect low-molecular-weight substances, most often in clinical practice for the determination of drugs [7,15,35] or toxicants [36,37]. A sufficient number of publications have been published on the determination of low-molecular-weight analytes by the FPIA method [7,13,14]. Small molecules (for example, drugs or hormones) do not change polarization upon binding, as their rotation remains rapid. Only competitive displacement of the labeled analog from the antibody allows for the detection of a signal inversely proportional to the analyte concentration. This ensures high specificity, but suffers from a narrow dynamic range, requiring prior knowledge of the expected concentration.

For detecting large biomolecules (proteins, antibodies, enzymes), the direct binding format is employed, where FP signal increases upon binding of a fluorescently labeled ligand to the target (Table 3). Unlike competitive formats (used for low-molecular-weight substances), the size of the recognition reagent becomes critical. Full-length monoclonal antibodies (IgG, ~150 kDa) yield only a modest polarization change (ΔmP) when binding to a large protein target because the hydrodynamic radii of the free antibody and the complex are similar. Therefore, smaller binders—nanobodies (VHH, 12–15 kDa) or Fab fragments (~50 kDa)—are preferred [17,24,25]. For example, when a nanobody binds to an 80-kDa protein (e.g., lactoferrin) or a 76-kDa protein (transferrin), the rotational correlation time of the complex increases, translating into ΔmP values of 40–50 mP and enabling limits of detection in the 1–10 µg/mL range in buffer solutions [23,24]. To provide a structured overview, Table 3 summarize the recommended FPA formats and performance characteristics for the major classes of biomarkers.

The main problem is the viscosity and optical density of biological fluids. For nucleic acids, hybridization requires strict temperature control, and fluorescence quenching is often observed in complex matrices (serum, blood). Viral particles, on the other hand, produce a high initial FP signal, but their detection suffers due to aggregation and nonspecific adsorption on the cuvette walls. A direct format with nanobodies for proteins or a competitive format for small molecules is recommended. A key criterion is minimizing sample pretreatment (hemolysis and lipidemia are critical). Plate readers with automatic G-factor calculation are suitable.

*Matrix effects and conjugation hurdles*

In real clinical specimens (serum, plasma, saliva), endogenous fluorophores (bilirubin, porphyrins) and light-scattering particles (lipoproteins, chylomicrons) generate substantial nonspecific background. In a homogeneous FP format (no washing step), this background is indistinguishable from the true polarization signal. This is particularly problematic for direct binding assays without prior analyte extraction: even with modern benchtop polarimeters, sensitivity in serum drops 3- to 5-fold compared to buffer and inter-sample CVs often exceed 15%. Several effective approaches have been proposed that can be combined depending on the matrix and analyte type. The simplest and most commonly used method is sample dilution with buffer (e.g., 1:10 or 1:20). This reduces the concentration of all interferents, including endogenous chromophores, proteins, and lipids. For example, when determining α-amylase in serum, the authors used a 10-fold sample dilution and automatic correction using a “blank” sample to reduce matrix interference. This enabled them to achieve a coefficient of variation of <6% in the concentration range of 10–500 U/L [19]. However, significant dilution can dramatically decrease the signal and increase statistical error. Classic studies on the determination of drugs in serum (e.g., for phenytoin or digoxin) have shown that a 1:10 dilution followed by correction using a calibration curve on the same matrix provides acceptable precision (CV < 5%) while maintaining sensitivity [38,39]. Large suspended particles (chylomicrons, cellular debris) can be removed by centrifugation at 10,000–15,000× *g* for 5–10 min or filtration through a 0.22 μm filter, which significantly reduces light scattering. This step is mandatory when working with lipemic or hemolyzed samples. Some protocols additionally use ultrafiltration to remove high-molecular-weight proteins if they are not involved in the analysis [38]. Switching to dyes excited in the red and near-infrared regions (650–900 nm) allows for the almost complete elimination of autofluorescence of endogenous components, as biological tissues and fluids exhibit virtually no fluorescence in this range. For example, cyanine dyes Cy5, Cy7, and Alexa Fluor 647, 680 are successfully used in serum fluorescence assays and even for intravital diagnostics. It has been shown that the matrix contribution is reduced by an order of magnitude when using such probes compared to fluorescein [40]. A study of vancomycin drug monitoring showed that replacing fluorescein with Cy5 and single-pass filtration of serum reduces the systematic error from 15% to <3% compared to HPLC [41]. To account for viscosity and background fluorescence, internal standards are often used, or mathematical correction methods are developed, such as subtraction of the signal of a blank sample (without analyte) or the use of dual-channel detection. Using an internal standard, the possibility of determining drugs in human blood plasma has been demonstrated [42], where the authors proposed a viscosity calibration curve using sucrose solutions and subsequent mathematical subtraction.

Moreover, covalent labelling of nanobodies or peptide substrates with fluorophores (e.g., FITC, Cy5) can alter the binding site conformation, potentially reducing the affinity constant and, in cases of over-labelling, may even abolish binding entirely [43]. Consequently, each labelled reagent batch may require rigorous optimization and quality control, a step that can limit reproducibility across laboratories and complicate point-of-care deployment, especially when sample pre-treatment (dilution, filtration, or extraction) is needed.

**Table 3:** Representative performance parameters of FP-based assays for selected biomarker classes. Values are specific to the analyte, matrix, and detection format indicated; they should not be extrapolated to all members of the class without independent validation.

Biomarker Class	Representative Analyte (Matrix)	FP Format	Recognition Element	LOD (Reported)	Detection Range	Time (Incl. Prep.)	Key Matrix Limitation	Ref.
**Small molecules**	Cortisol (serum)	Competitive FPIA	Anti-cortisol antibody	1.5 ng/mL (~4.1 nM)	1.5–100 µg/dL	15 min	pH sensitivity; cross-reactivity with prednisolone	[44]
	Theophylline (serum)	Competitive FPIA	Anti-theophylline antibody	0.5 µg/mL (~2.8 µM)	0.5–40 µg/mL	10 min	Bilirubin interference at >5 mg/dL	[45]
**Large proteins**	Lactoferrin (human milk)	Direct FP with nanobody	FITC-labelled VHH (15 kDa)	0.5 µM	0.5–10 µM	5 min	No data in serum; viscosity effect not assessed	[23]
	C-reactive protein (serum)	Direct FP with Fab fragment	Fab-Alexa Fluor 647 (~50 kDa)	0.8 nM	1–200 nM	20 min (dilution 1:10)	Serum dilution reduces background; correlation with ELISA r = 0.92	[17]
**Nucleic acids**	HIV-1 DNA (buffer)	Competitive hybridization with AuNP amplification	Fluorescein-labelled probe	73 pM	0.1–10 nM	60 min	Only tested in buffer; not validated in plasma	[46]
	HBV DNA (serum)	Asymmetric PCR + FP assay	FITC-labelled probe	10 copies/µL (~17 aM)	10–10^6^ copies/µL	2.5 h (incl. PCR)	Requires DNA extraction; inhibition by hemoglobin	[47]
**Viral particles**	SARS-CoV-2 spike (buffer)	Direct aptamer binding	Cy5-aptamer (51-m)	0.5 nM	0.5–50 nM	15 min	Aggregation and non-specific binding in saliva	[48]
**Cells**	Breast cancer cells (culture)	Methylene blue accumulation	–	Not applicable (qualitative FP increase)	–	5 min	Requires fresh unfixed cells; not quantitative	[49]

*Clinical applications, Limitations, and Comparison with Alternative Methods*

For high-molecular-weight biomarkers, the direct FP format relies on recognition reagents with a small hydrodynamic volume. As discussed above, nanobodies (~15 kDa) are particularly effective. Their small size ensures that binding to a protein target (e.g., lactoferrin, 80 kDa; transferrin, 76 kDa) results in a 5- to 7-fold increase in rotational correlation time compared to a full-length antibody, translating into a significantly larger ΔmP and lower detection limits. However, even with nanobodies, the polarization increases upon binding to targets > 100 kDa becomes marginal (ΔmP < 40 mP), because the rotational correlation time of the complex approaches the upper detection limit of typical polarimeters. Consequently, FP is ill-suited for discriminating between isoforms (e.g., free vs. bound protein) due to the small difference in molecular weight between such variants, nor is it optimal for detecting intact virions without enzymatic digestion.

In Table 4, we compare widely used biomarker discovery methods with the FP method. This comparative analysis shows that FP occupies a unique niche between the speed, low cost, and simplicity of lateral flow assay (LFA) and the high sensitivity and complex work flows of ELISA, PCR, and CLIA. FP’s most distinctive feature is its homogeneous format—it requires no washing or separation steps. This contrasts sharply with ELISA and CLIA, which require numerous incubations and washing steps, increasing run time and introducing technical variability. The absence of these steps allows FP to obtain results in less than 5–15 min, making it comparable to LFA in speed. However, unlike most LFAs, FP provides fully quantitative data over a continuous dynamic range, a significant advantage for accurately monitoring drug concentrations or biomarker levels. By its nature, FP’s sensitivity is limited compared to PCR and CLIA. For low-concentration targets—such as early-stage viral RNA or cytokines with concentrations below pg/mL—PCR and CLIA remain the methods of choice. However, for analytes with medium and high concentrations, particularly small molecules (drugs, hormones) and biomarkers present in concentrations from ng/mL to µg/mL, the sensitivity of FP is quite sufficient. This makes it ideal for therapeutic drug monitoring (TDM), where target concentrations are typically in the range of 1–100 µg/mL and rapid results are crucial clinically. Although FP requires minimal sample preparation, it remains more sensitive to matrix interferences (viscosity, autofluorescence, light scattering) than heterogeneous assays such as ELISA and CLIA, which can wash out interferences. This limitation can be mitigated by simple dilution, filtration, or the use of near-infrared fluorophores, as discussed in the previous section. PCR requires the most extensive sample preparation (nucleic acid extraction), limiting its use to well-equipped laboratories unless integrated into cartridges. FP assays are moderately priced and highly automated—as demonstrated by the Abbott TDx and AxSYM platforms, which enabled the processing of hundreds of samples per day without operator intervention. In contrast, PCR instruments and CLIA-compliant luminometers require large capital investments, although ELISA and LFA remain the most accessible for small volumes or single-use applications. LFAs are superior in cost and simplicity, but typically sacrifice quantitative accuracy and sensitivity. FP assays are clinically mature and FDA-approved for a wide range of small-molecule drugs (antiepileptic drugs, antibiotics, immunosuppressants) and hormones (cortisol, T3). Its expansion to large protein biomarkers using nanoantibodies is still in the implementation phase, and proof-of-concept studies are showing promising results. However, FP analysis is not intended to replace PCR, ELISA, or CLIA, but rather to complement them—especially in scenarios where speed, simplicity, and moderate sensitivity are more important than ultra-low detection limits.

Nevertheless, when combined with microfluidics for automated sample handling and NIR fluorophores to reduce background, FP assay may find valuable niche applications in point-of-care testing (POCT) for acute conditions where speed and simplicity outweigh the need for ultra-high sensitivity [47,48]. Emerging paper-based and smartphone-integrated FP platforms further lower the barrier to deployment in resource-limited settings, provided that matrix-matched calibration and robust G-factor correction is implemented. In conclusion, the choice of FPIA format—competitive vs. direct, and IgG vs. nanobody—must be guided by the molecular weight of the target, the available sample volume, and the required sensitivity. While FPIA is a mature technique for small molecules, its extension to high-molecular-weight biomarkers demand careful optimization of the recognition element, the fluorophore, and the sample pre-treatment protocol. With these considerations addressed, FPIA remains a powerful, rapid, and cost-effective tool for modern clinical diagnostics. Nevertheless, when combined with microfluidics and near-infrared fluorophores (which reduce autofluorescence background), FP may find niche POCT applications in rapid semi-quantitative testing for acute conditions where speed outweighs absolute accuracy [56,57].

Fluorescence polarization offers a convenient homogeneous format for detecting a wide range of biomarkers, yet its clinical translation is hampered by matrix interference, labelling artefacts, and lack of standardization. For high-molecular-weight targets, nanobodies and antibody fragments are preferable, but each assay requires case-by-case optimization and thorough validation against reference methods on large clinical cohorts. Currently, only competitive FPIA for small molecules (hormones, drugs) has reached the level of routine laboratory testing; other applications should be viewed as promising but not yet ready for broad clinical practice.

*FP signal-amplification*

However, the change in polarization signal directly depends on the change in molecular mass. If the analyte itself is small, even after binding to the probe, it will not become large enough to significantly slow its rotation. The signal change will be too small to be reliably detected. The same thing happens when the target is very small: there are simply not enough bound complexes to generate a noticeable signal.

Signal amplification strategies are designed to artificially increase the “effective mass” of the detected complex or to greatly enhance the signal itself. The comparative summary presented in Table 5 highlights that the choice of an amplification strategy for FP assays inevitably involves tradeoffs. Nanoparticle-based approaches offer rapid and robust signal enhancement in a homogeneous format, but their application is hampered by light scattering interference and limited compatibility with complex biological matrices. In contrast, nucleic acid amplification provides the highest sensitivity, but at the expense of prolonged assay time, increased complexity, and susceptibility to enzymatic inhibitors. Protein aggregation, while conceptually straightforward, suffers from reproducibility issues and matrix effects. Emerging strategies such as target recycling or conformational switching are promising, but their versatility and practical robustness remain to be fully validated. Therefore, the most suitable method should be carefully selected based on the specific analytical requirements, sample type, and available instrumentation.

Thanks to these advances, the sensitivity of methods increases by orders of magnitude: the detection limit can be reduced to low concentrations, which is more sensitive than traditional methods. Thus, all these complex strategies—from the use of nanoparticles to cascade enzymatic reactions—are urgently needed. They transform fluorescence polarization from a method limited to large molecules into a universal tool for the ultrasensitive detection of a wide variety of molecules, including small drugs, cancer marker proteins, and microRNAs, opening up new possibilities for early diagnostics and biomedical research.

### 3.1. Infectious Disease Diagnosis by FP Assay in Humans

Detection of infectious diseases using FP is based on the identification of various pathogen-associated biomarker targets. These targets include: (1) antibodies, since the immune system produces specific antibodies when a pathogen enters the body: FP analysis enables rapid and accurate detection of such antibodies in biological fluids (e.g., serum, milk, or saliva); (2) antigens and nucleic acids: the method is also applicable for the direct detection of bacterial and viral antigens, as well as for the identification of specific DNA and RNA sequences of pathogens.

#### 3.1.1. Antibody Determination by FP Assay

Immunoglobulins (also known as antibodies) are high-molecular-weight glycoproteins produced by plasma cells in response to antigenic stimulation. Their concentrations and isotype distributions are altered in infections, autoimmune diseases, allergic disorders, immunodeficiencies, and certain malignancies, making them widely used biomarkers for the diagnosis, prognosis, and monitoring of these conditions. Fluorescence polarization assay has found widespread application in human infectious disease diagnostics, from serological testing for viral hepatitis to rapid point-of-care assays during pandemics.

FP assay offers several advantages for clinical diagnostics: it is exceptionally rapid (results within minutes), highly sensitive, requires no complex sample preparation, allows full automation, and needs only a few microliters of sample. The method is homogeneous (“mix-and-measure”), eliminating washing and separation steps. However, these benefits must be weighed against known limitations, notably the susceptibility to optical interference from endogenous fluorophores (bilirubin, porphyrins) and light-scattering particles (lipoproteins) in undiluted serum, which can reduce sensitivity and reproducibility. Sample pre-treatment (dilution or filtration) is often necessary for reliable measurements in real-world clinical specimens.

*FP assay of antibody detection of bacterial infections*

FP assay has proven effective in detecting antibodies to bacterial contaminants, particularly against Gram-negative bacteria [63]. Due to the structural features of the cell surface and the presence of antigenic lipopolysaccharides in each bacterial species, researchers have been able to isolate specific oligo- or polysaccharides and obtain their fluorescently labeled derivatives. Using these derivatives as recognition reagents, FP assay has been successfully applied to the diagnosis of brucellosis and bovine tuberculosis in animals (the sensitivity and specificity were 95.5% and 99.0% for brucellosis and 92.9% and 98.3% for tuberculosis, respectively [64,65]). FP assay has been successfully applied to diagnose brucellosis in humans [66,67]. Using fluorescently labeled lipopolysaccharide (O-polysaccharide) as a recognition element, FP assay detects anti-*Brucella* antibodies in human serum with sensitivity and specificity comparable to ELISA but with much faster turnaround time [68] (Table 6).

*FP antibody detection of viral infections*

**Figure 4:**
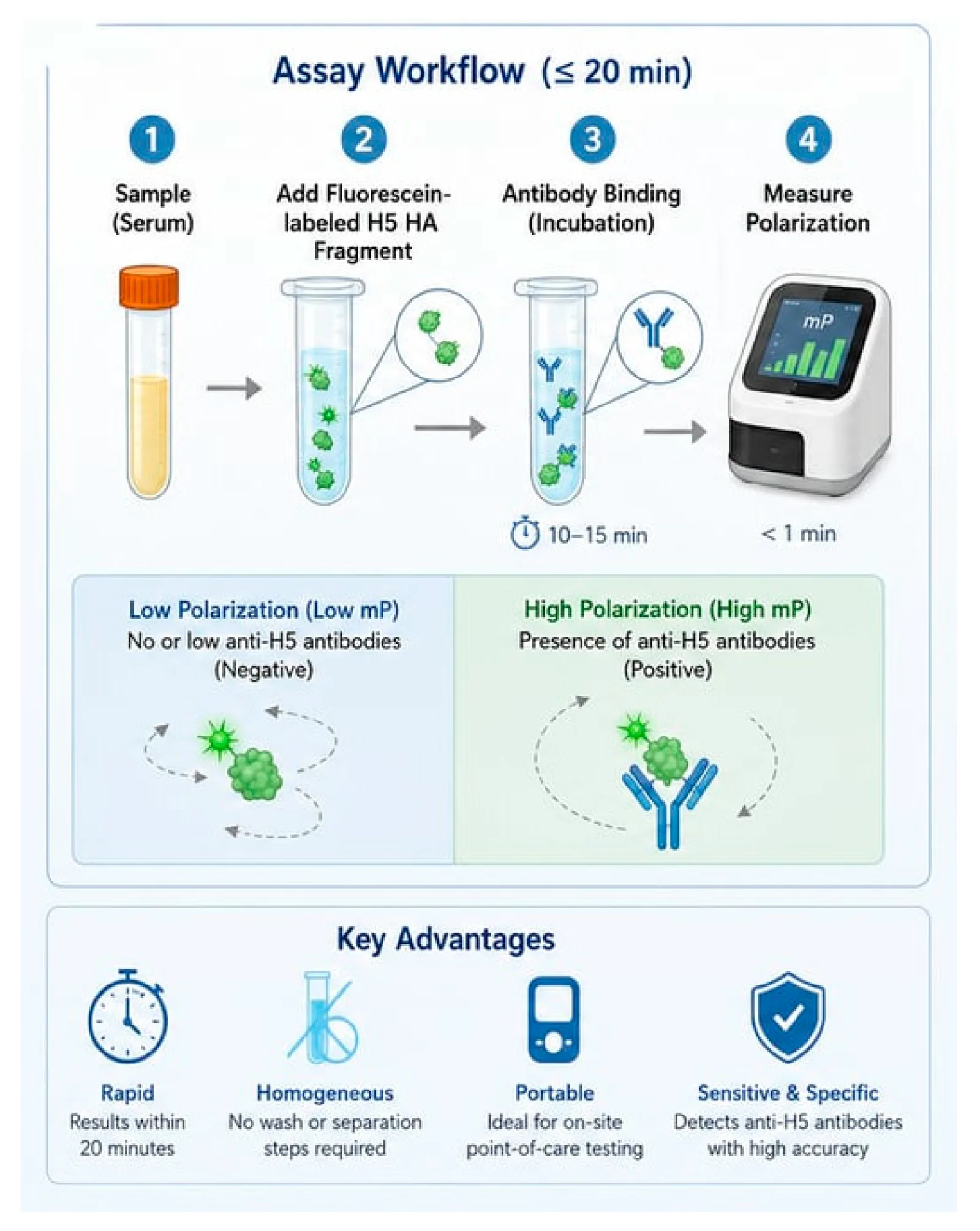
Fluorescence polarization assay for anti-H5 antibody detection using a fluorescein-labeled antigen fragment as tracer.

A promising non-competitive fluorescence polarization assay format uses fluorescein-labeled antigen fragments as tracers, which increase polarization upon binding to specific antibodies [81]. The method is based on the classic fluorescence polarization principle: a small fluorescein-labeled antigen fragment, freely rotating in solution, exhibits low polarization. Upon binding to a large antibody molecule present in serum, its rotational motion slows markedly, producing a measurable increase in polarization (Figure 4). In this assay, a fluorescein-labeled fragment of the H5 hemagglutinin protein serves as the tracer, while anti-H5 antibodies in the sample act as the binding partners. If the patient was previously infected with the H5 avian influenza virus, their immune system will have produced anti-H5 antibodies. In the fluorescence polarization assay, these antibodies bind to the fluorescein-labeled H5-HA fragment used as the tracer. The resulting antigen–antibody complex has a much larger molecular size than the free HA fragment and therefore rotates more slowly in solution. This decrease in rotational diffusion generates a significant increase in the fluorescence polarization signal (Figure 4). Because the method is homogeneous and relies solely on changes in rotational mobility, no separation of bound and free tracer is required. The procedure is simple, rapid, and well suited for on-site antibody detection using portable FP instrumentation. The total analysis time was within 20 min, which is significantly shorter than the time required by conventional ELISA-based devices.

The COVID-19 pandemic has provided a powerful impetus for the development of FP methods (Table 6). A noncompetitive FP assay has been proposed. It uses a recombinant SARS-CoV-2 S protein as a probe, specifically its receptor-binding domain (RBD), labeled with a fluorescent dye, such as HiLyte Fluor 647 (F-RBD). When serum is added to a sample, if antibodies to SARS-CoV-2 are present, they bind to F-RBD. This leads to the formation of a large complex whose rotation slows, and the FP signal increases. The higher the signal, the more antibodies are present in the sample. This method quantitatively detects antibodies to SARS-CoV-2 in human serum in just 20 min, using only 0.25 µL of sample. The test is highly accurate, with an area under the receiver operating characteristic (AUC) of 0.965. The method is based on a fluorophore-labeled recombinant viral RBD protein [70]. When binding to the antibody, its rotation slows, increasing the signal.

HIV infection is routinely diagnosed by antibody detection; FP formats have been developed for rapid HIV serology [46,62]. Regarding viral hepatitis, FP-based detection of anti-hepatitis B core antigen (anti-HBc) and anti-hepatitis C virus (HCV) antibodies has been demonstrated, providing a rapid alternative to conventional ELISA [47,82].

*FP assay for determination of the neutralizing antibodies—Two Distinct Scenarios*

Antibody screening is important because it not only detects an immune response but also identifies potentially dangerous inhibitory antibodies that can impact the course of the disease and the effectiveness of treatment. Another important reason for screening antibodies in a patient’s blood is that when treating certain chronic diseases, such as multiple sclerosis with interferon-beta, the patient’s body may begin to produce antibodies to the drug. Some patients who do not respond to therapy actually develop neutralizing antibodies to the drug. In such cases, the level of neutralizing antibodies directly correlates with the treatment response. In patients with clinically significant neutralizing antibody titers, therapy may be ineffective. Detecting these antibodies helps physicians decide whether to change the drug, which is especially important for expensive biologic drugs. A similar problem is observed when treating autoimmune diseases such as rheumatoid arthritis or Crohn’s disease with biologic drugs (e.g., TNF-α inhibitors). Detecting neutralizing antibodies (those capable of blocking the virus) is crucial for assessing herd immunity and vaccine effectiveness. Neutralizing antibodies not only bind to the pathogen but directly block its ability to penetrate host cells, thereby preventing infection [83,84]. Their determination plays a key role in assessing the strength of post-vaccination or post-infection immunity. The key difference between conventional antibodies, which simply bind to the target, and inhibitory (neutralizing) antibodies is that the latter do not simply signal the presence of a pathogen, but suppress its biological activity [85]. This is why their detection is of particular, and sometimes decisive, clinical importance. A high-throughput fluorescence-based assay for the quantitative determination of neutralizing antibodies has been developed. It is based on measuring the ability of antibodies from a patient’s serum to block the interaction between the viral RBD protein and the ACE2 receptor on a human cell [85]. When this key step in viral entry is blocked, the specific fluorescent signal changes. This assay has demonstrated results comparable to the “gold standard”—the plaque neutralization assay (PRNT), but is significantly faster, simpler, and suitable for mass automated screening [85]. This makes it indispensable for assessing protective immunity in large populations and in vaccine development.

#### 3.1.2. FP for the Determination of Nucleic Acids of Human Pathogens

The FP assay has also been successfully used to detect nucleic acids. This enables genetic diagnostics, identifying pathogens themselves and their antibiotic resistance genes. Various approaches are currently used to diagnose human viral infections. To increase the sensitivity of SARS-CoV-2 virus detection in the early stages, FP assay was successfully combined with advanced molecular diagnostic methods. A portable system was developed that combines isothermal amplification of viral nucleic acids and the CRISPR/Cas12a system. Cas12a is activated upon detection of target viral RNA and begins non-specifically cleaving fluorophore-labeled single-stranded DNA probes. Studies have shown that this system can achieve sensitivity of up to 100 femtomolar (fM) without prior amplification. This method is crucial for the early detection of infection, when the amount of virus in the body is still very low [60].

For the human immunodeficiency virus (HIV), detection methods include antibody tests, combined antigen/antibody tests, and nucleic acid tests. Hepatitis B virus (HBV) is identified using three main markers: HBV surface antigen (HBsAg), antibodies to HBsAg (anti-HBs), and antibodies to the core antigen (anti-HBc). However, antibody detection is only possible approximately one month after infection, making them unsuitable for early diagnosis. Viral nucleic acids appear as the earliest indicators after viral entry [62].

A fluorescence polarization (FP)-based platform for screening HIV and HBV has been developed. One study described a method for detecting HIV DNA based on T7 exonuclease amplification with target recycling in combination with graphene oxide (GO); the detection limit was 38.6 pM [46,62]. Another study used dendritic-modified gold nanoparticles to enhance the FP signal, achieving a detection limit of 73 pM for HIV DNA [46].

The hepatitis B virus is classified into eight genotypes (A to H) based on differences in genomic sequences. Patients infected with different HBV genotypes respond differently to drug therapy and have different risks of disease relapse and adverse effects. A method using FP assay with asymmetric PCR and hybridization was developed to identify HBV genotypes A to D [47]. Thus, the FP assay-based platform not only quantifies HBV DNA but also determines the genotype, which is crucial for choosing treatment strategies and predicting disease severity.

Recent advances in fluorescence polarization assay have prioritized its combination with amplification platforms—particularly PCR and loop-mediated isothermal amplification (LAMP). Such synergy affords ultra-high sensitivity, allowing the detection of as little as a single genomic copy or one bacterium in a specimen. Numerous reports confirm that FP assay is effective for a broad spectrum of nucleic-acid pathogens, encompassing both DNA and RNA viruses and bacteria.

### 3.2. FP Assays for High-Molecular-Weight Biomarkers

Proteins and enzymes represent a diverse class of high-molecular-weight biomarkers that provide critical information for disease diagnosis, prognosis, and therapeutic monitoring. Their detection by fluorescence polarization (FP) faces a fundamental challenge: the change in molecular volume upon binding of a fluorescently labelled probe to a large target is relatively small, resulting in a modest increase in polarization signal. To overcome this limitation, several strategies have been developed: (i) the use of fluorescently labelled small recognition elements (nanobodies, affibodies, aptamers) that undergo a significant relative size increase upon binding; (ii) the use of mass enhancers (nanoparticles, polymers) to amplify the polarization change; (iii) competitive displacement formats where a large labelled analyte is displaced by a smaller unlabeled one; and (iv) enzymatic signal amplification. This section critically evaluates these strategies, with particular emphasis on the quantitative relationship between reagent size and assay sensitivity.

#### 3.2.1. Protein Determination by FP Assay

The direct FP assay format is used to detect high-molecular-weight biomarkers (antibodies, proteins, viral particles) [8]. A high-molecular-weight biomarker binds to a fluorescently labeled antigen with a significantly lower molecular weight, slowing the rotational diffusion rate of the fluorophore and increasing the FP signal.

FP aptasensors for disease detection are rapid, homogeneous, and sensitive diagnostic tools that use short, fluorescently labeled DNA/RNA aptamers to bind cancer biomarkers. They work by measuring changes in the rotational speed of these labeled aptamers when they bind to larger targets—such as proteins or cancer cells—significantly increasing the polarization signal.

Prostate-specific antigen (PSA) is a key tumor marker for prostate cancer. To increase the sensitivity of FP assay for PSA detection, approaches using nanotechnology are being actively developed. In [72], the use of a copper-containing metal–organic gel (Cu-MOG) as a platform for fluorescence anisotropy signal enhancement was demonstrated. A fluorescently labeled aptamer (PA) is immobilized on the Cu-MOG surface via electrostatic interactions and π–π stacking, resulting in a significant increase in the molecular volume of the complex and, consequently, an increase in the anisotropy signal. In the presence of the target PSA, the aptamer specifically binds to the antigen, leading to dissociation of the complex and release of the aptamer from the gel surface, resulting in a decrease in the anisotropy signal. This strategy provides a linear dynamic range from 0.5 to 8 ng/mL with a detection limit of 0.33 ng/mL. This approach marks an important step in the application of metal–organic materials for biomolecular analysis and disease diagnostics. In addition to PSA detection, the FP method is actively developing toward the simultaneous (multiplex) analysis of several tumor markers [33]. In a study on the use of quantum dots (QDs) in FP, the authors demonstrated the possibility of simultaneously detecting alpha-fetoprotein (α-AFP) and carcinoembryonic antigen (CEA). The use of quantum dots with a long fluorescence lifetime circumvents the limitations of traditional fluorophores and expands the range of analyzed high-molecular compounds. This approach is particularly relevant for cancer diagnostics, where the use of marker panels significantly increases the diagnostic value of the analysis compared to the detection of a single biomarker.

Thus, modern developments in FP for PSA detection are aimed at creating highly sensitive, homogeneous, and rapid systems that could become an effective alternative to traditional heterogeneous immunoassay methods. The introduction of nanotechnology (metal–organic gels, nanoparticles) and the use of aptamers as receptor molecules makes it possible to overcome the limitations of classical FP analysis associated with insufficient sensitivity in the detection of high-molecular biomarkers.

In addition to aptamers, nanobodies (single-domain antibodies, VHH antibodies, or sdAbs) are promising recognition reagents. These are miniature recombinant antibodies consisting of a single functional domain—the variable fragment of the heavy chain of antibodies, which are naturally found in members of the camelid family (camels, llamas, alpacas) and some species of sharks [86]. Unlike classical monoclonal antibodies, which consist of two heavy and two light chains, nanobodies have a molecular mass of only about 12–15 kDa, which is approximately 10 times less than that of traditional antibodies. Due to their unique physicochemical properties, nanobodies offer a number of important advantages: high stability, ease of production, the ability to recognize hidden epitopes, and the ability to bind to hard-to-reach regions of target molecules (conformational epitopes) inaccessible to classical antibodies. A series of studies was conducted on the creation and application of fluorescently labeled nanobodies for the highly sensitive and specific determination of biological markers in human physiological fluids using FP assay for the determination of major proteins in human blood plasma.

Human lactoferrin (hLF) is a multifunctional glycoprotein with immunomodulatory, anti-inflammatory, antibacterial, antiviral, and antioxidant properties. It is also a marker of oncological diseases [23,87]. Fluorescently labeled recombinant nanobodies were used for the quantitative determination of hLF, enabling the efficient determination of relatively large molecules such as lactoferrin using FPIA [23,87]. Another human plasma protein, transferrin (Tf), exists in two forms: iron-containing (holo-transferrin, holo-Tf) and iron-free (apo-transferrin, apo-Tf). The quantitative ratio of these forms is an important biochemical marker for diseases associated with iron deficiency or excess in the body. A study [24] demonstrated the feasibility of detecting two forms of transferrin in human physiological fluids using nanobodies capable of recognizing conformational changes in transferrin associated with the addition or loss of an iron ion, enabling the differential detection of holo-Tf and apo-Tf.

The resulting fluorescently labeled nanobodies against human antibodies (IgG and IgA) were used for the rapid and specific detection of immunoglobulins G and A in human serum. The researchers obtained and characterized fluorescently labeled nanobodies against human IgG and IgA (FITC-anti-IgG and FITC-anti-IgA). The dissociation constants (KD) of the nanobody–antigen complexes were determined, confirming the high affinity of the resulting immunoreagents for their targets. The FPIA method allows for the determination of IgA levels in human serum in the range of 35–120 μg/mL and IgG levels in the range of 75–260 μg/mL. Thus, fluorescently labeled recombinant nanobodies are a highly effective tool for the rapid, sensitive, and specific determination of protein concentrations and their conformations in human biological fluids. The developed methods open up broad prospects for clinical diagnostics, immune system monitoring, and the assessment of iron metabolism disorders.

Another type of antibody that may be promising for use as recognition reagents in FPIA are affibodies. They are small, engineered proteins selected to bind to target molecules of interest. These small affinity probes consist of 58 amino acids and form a structure of three α-helices [73]. Due to their small size (~6.5 kDa), affibodies outperform their monoclonal antibody counterparts in accessing and detecting target molecules on extracellular vesicles (EVs). Thus, they are poised to become the next generation of affinity probes. These affibodies have been used to recognize HER2 (a cancer biomarker) and detect HER2 receptors on HER2-positive EVs [73,88]. Small HER2 affibody molecules have been shown to outperform HER2 monoclonal antibodies by an order of magnitude in accessing HER2 receptors on EVs. Furthermore, HER2 affibody molecules outperform HER2 antibodies in detecting and quantifying HER2-positive EVs in both buffered saline and human plasma using colorimetric and fluorescence polarization-based assays [73].

#### 3.2.2. FP Assay for Determination Enzyme Activity

Changes in enzyme activity (e.g., proteases) can also serve as disease markers. Natural components with a high molecular weight are often used as substrates [77,89]. After the introduction of a fluorescent dye, these components produce a high FP signal. Enzymatic cleavage produces small fragments, resulting in a decrease in the FP signal over time. A significant advantage of methods based on the phenomenon of fluorescence polarization is the possibility of direct and continuous monitoring of the enzymatic reaction in real time.

**Figure 5:**
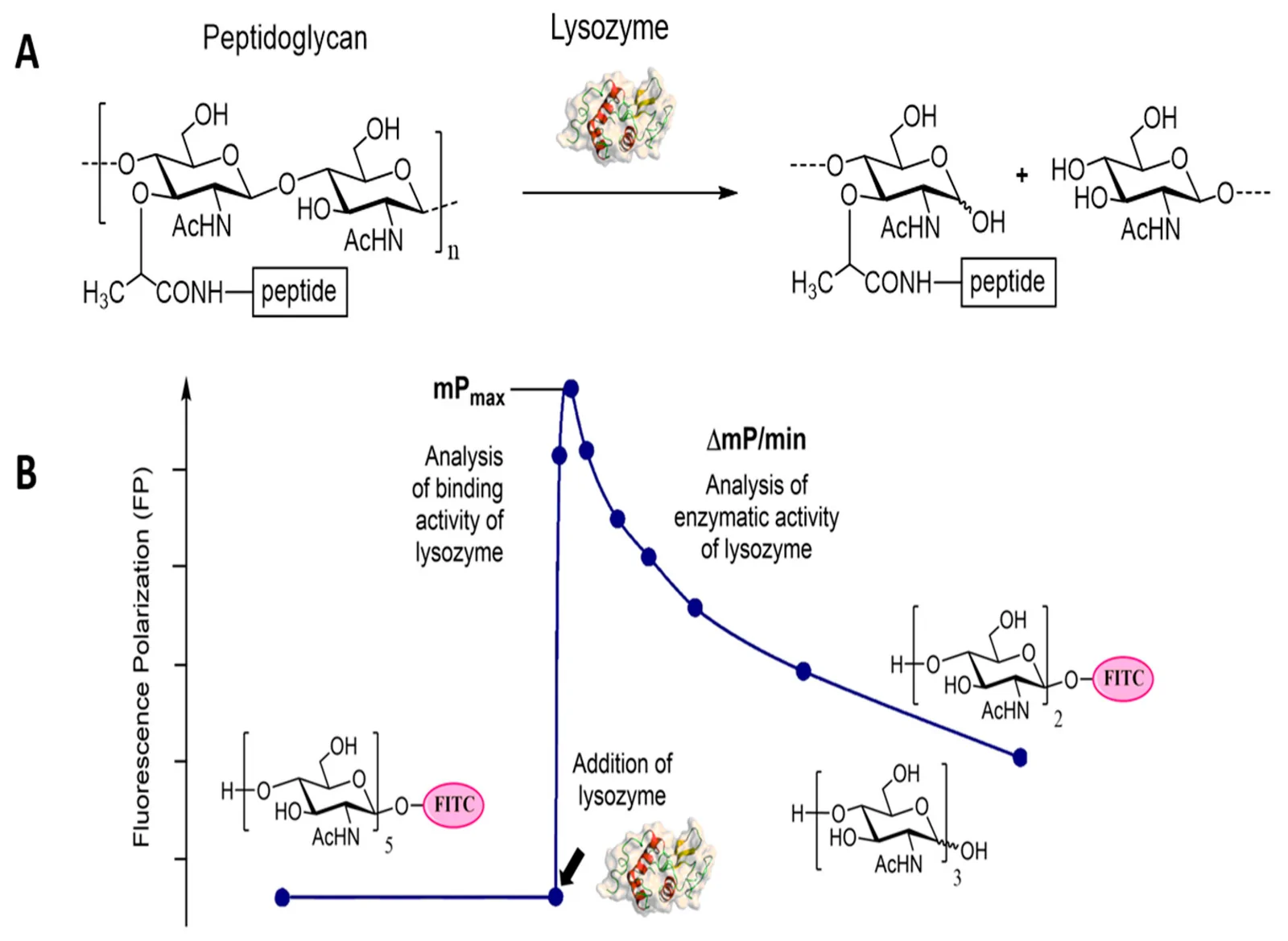
Hydrolysis by lysozyme of fluorescently labeled substrates: (**A**) natural peptidoglycan and (**B**) synthetic oligosaccharide substrates.

The first FP assay was used to analyze lysozyme and relied on fluorescently labeled peptidoglycan [77]. Lysozyme plays a key role in antibacterial defense by hydrolyzing the peptide glycan of the bacterial cell wall. Traditional methods for analyzing its activity, such as turbidimetry (based on the turbidity of a suspension of *Micrococcus lysodeikticus* bacteria), have significant drawbacks: they are labor-intensive, poorly reproducible, and use non-standardized reagents (living bacteria) [67]. At the time, FP assay was revolutionary, offering an alternative to traditional turbidimetry. However, a natural fluorescent substrate was obtained from the cell wall of the bacterium *Micrococcus lysodeikticus*, purified, and then covalently labeled with fluorescein isothiocyanate. Reaction mechanism and signal detection: The key principle of the method is based on the change in the physical state of the substrate during hydrolysis. Initial state: Intact FITC-labeled peptidoglycan is a large polymer that rotates slowly in solution, resulting in highly polarized fluorescence (high *p*-value) (Figure 5A). Hydrolysis by lysozyme: Lysozyme cleaves glycosidic bonds in peptidoglycan, cutting long polymer chains into shorter fragments. The most common mechanism of lysozyme action is the hydrolysis of β-(1→4)-glycosidic bonds between N-acetylmuramic acid and N-acetyl-D-glucosamine residues. The method demonstrated excellent specificity and sensitivity. Changes in the FP signal were shown to be specific to the action of lysozyme, while other hydrolytic enzymes (such as α-mannosidase, proteases, and RNase) had no effect on this parameter. A change in the polarization signal was detected even with the addition of lysozyme at a concentration of 0.01 μg/mL. Furthermore, this method enables the determination of kinetic constants (apparent Km and Vmax) for lysozymes from different sources.

Although fluorescence polarization (FP) is not always applicable for direct observation of reactions with natural substrates, it becomes a powerful and informative tool when using properly designed synthetic substrates. A fundamental advantage of the FP method for studying enzymes using synthetic substrates with a well-defined structure is that fluorescently labeled chito-oligosaccharides of varying lengths (from tri- to pentasaccharides) are used instead of highly heterogeneous natural peptide glycan [79]. Depending on the length of the oligosaccharide chain, FP analysis allows for the simultaneous monitoring of two processes: the formation of an enzyme-substrate complex (the increase in polarization signal upon binding of the enzyme (lysozyme) to the labeled substrate is analyzed) and the hydrolysis of the substrate (Figure 5A), which leads to rapid rotation of the molecule and a sharp drop in FP signal (Figure 5B). The proposed protocol allows for the determination of human lysozyme with a detection limit of 0.3 μM and a complete analysis time of approximately 30 min. The method has been successfully tested for the determination of lysozyme activity in human tear fluid, demonstrating good correlation with the reference ELISA method.

Another example of the use of FP for determining enzymatic activity is shown using α-amylase as an example [89,90]. α-Amylase (EC 3.2.1.1) belongs to the glycoside hydrolase family and hydrolyzes starch by randomly cleaving α-1,4-glycosidic linkages, producing maltotriose, maltose, glucose, and limit dextrins from both amylose and amylopectin. In mammals, this enzyme is a key digestive hydrolase. Elevated α-amylase levels in humans are clinically associated with salivary gland trauma, mumps (due to parotid inflammation), pancreatitis, and renal failure [19,20,89,90,91]. In research and drug discovery, there is a strong demand for simple, direct, and automation-compatible assays to measure α-amylase inhibition.

The development of FP for amylase determination has progressed from fundamental research to clinical implementation. This pioneered the concept of using fluorescence polarization for macromolecular hydrolases, including α-amylase. The key principle is the direct and continuous measurement of the drop in polarization during hydrolysis of a fluorescently labeled substrate [91]. The technology was then adapted for the quantitative determination of α-amylase in serum and urine using the Abbott TDx analyzer: initially, the method was based on the hydrolysis of FITC-labeled amylose; the decrease in polarization is directly proportional to the amylase activity [19]. Calibration with a panel of six human serum-based pancreatic amylase calibrators ensured reliable measurements. The coefficient of variation (CV) was less than 6%, and the reagents were stable in liquid form for a long time [91], making the method suitable for routine clinical diagnostics [19]. The method was later automated, confirming its potential for high-throughput screening [20]. Modern test (QuantiFluo™, QuantiFluo™ α-Amylase Inhibitor Screening Kit|BioAssay) is designed for screening amylase inhibitors. This kit is optimized for high-throughput screening using multiwell plates (up to 1536 wells), are non-radioactive, and provide results within an hour.

Thus, the use of fluorescence polarization in combination with chemically synthesized substrates offers a revolutionary approach to enzyme analysis that outperforms traditional methods in a number of ways. (1) Standardization: The elimination of natural biological substrates with poorly controlled composition in favor of strictly defined synthetic reagents ensures the highest reproducibility and standardization of the method. The use of synthetic substrates instead of natural ones will eliminate a fundamental limitation on the path to the implementation of FP in routine analytical practice and pharmacopeial analysis [92]. (2) Speed, simplicity, and the ability to use compact equipment allow results to be obtained within minutes. (3) Unlike many traditional enzymatic tests, FP assay with a properly selected substrate can simultaneously provide information on both enzymatic activity (catalytic constant) and protein concentration. Moreover, the analysis of the kinetics of polarization signal change (increase due to binding and decrease due to hydrolysis) provides the fundamental possibility of simultaneous measurement of several enzyme activities (for example, chitinase and lectin-like activity of lysozyme), which is crucial for its detection in complex biological systems [79,93]. (4) The homogeneous format and the possibility of miniaturization make the FP ideal for high-throughput screening (HTS) of drugs, allowing testing of thousands of potential inhibitors in a short time [12].

The diversity of high-molecular-weight biomarkers reflects the complexity of biological processes: they represent a large group of biopolymers that are naturally chemical compounds with large molecular weights, such as proteins and nucleic acids. Unlike low-molecular-weight metabolites, these macromolecules carry complex, multi-layered information about the body’s state. For example, nucleic acids (DNA/RNA) carry information about genetic predisposition, diagnosis, and prognosis, and can also indicate physiological conditions or infections. Proteins (PSA, lactoferrin), tumor markers (CA19-9, CEA), receptors (EGFR), antibodies, and cytokines have high prognostic value (they are most predictive in diagnosing complex diseases) and are widely used in diagnostics and therapy. The key advantages of FP-based enzyme assays—homogeneity, real-time monitoring, miniaturization, and the use of defined synthetic substrates—make them increasingly attractive for both drug discovery and clinical diagnostics. The development of fluorogenic substrates with improved quantum yields and longer lifetimes, combined with the use of nanoparticles for signal amplification, promises further improvements in sensitivity. Moreover, the compatibility of FP with microfluidic platforms and smartphone-based readers [39,40] suggests that enzyme activity assays will become increasingly accessible at the point of care.

### 3.3. Direct Detection of Viral Particles, Bacteria, and Cells by FP Assay

FP can be used to detect not only DNA but also entire bacterial cells. This is achieved using fluorescently labeled probe molecules that specifically bind to the bacterial surface. Unlike DNA analysis, where the signal arises from an increase in molecular weight during hybridization or amplification, FP uses probes that dramatically slow their rotation upon binding to the massive bacterial cell, resulting in a measurable increase in the polarization signal. In addition to detecting soluble biomarkers (antibodies, antigens, nucleic acids), FP assays enable the direct detection of entire pathogens—viral particles, bacterial cells. This approach enables rapid and quantitative assessment of microbial loads. It is important to note that some examples discussed below are presented for illustrative purposes only, to demonstrate the versatility of the FP principle across different target types; they are not part of a comparative analysis and do not imply that all mentioned applications are exclusively validated in human samples unless explicitly stated.

*Viral Particles and Bacterial Cells Detection*

The main strategy involves the use of highly specific probes that bind to a target on the cell surface:

1. Antibodies and their fragments (Fab fragments, nanobodies). A FPIA method for detecting Salmonella spp. cells is described. The method involves adding antiserum (with antibodies to Salmonella O-antigens) and a fluorescently labeled O-polysaccharide (tracer) to a test tube. In the absence of bacteria, the tracer binds to the antibodies, and the FP signal increases. When Salmonella cells are added, they compete with the tracer for antibodies → the tracer remains free and polarization decreases. A decrease in polarization indicates the presence of cells [63].
2. Peptides. A peptide for Edwardsiella tarda (an opportunistic pathogen causing intestinal infections and wound inflammation in humans) was identified, and an approach for sensitive bacterial detection using a fluorescein-labeled peptide was tested. Fluorescent peptide FP analysis enabled detection of E. tarda in the range from 5.2 × 10^3^ to 2.1 × 10^5^ cells [94].
3. Aptamers. Aptamers are a promising tool for infection detection. Aptamers—synthetic single-stranded DNA or RNA capable of specifically binding a target—serve as an alternative to antibodies. In a non-competitive FP assay with aptamers (Aptamer-based FP assay), binding of a fluorescently labeled aptamer to a viral protein (e.g., the SARS-CoV-2 spike protein) also leads to increased polarization. This approach allows for rapid and inexpensive detection of various virus variants. A particularly noteworthy study successfully used aptamer-based FP assay to detect inactivated whole SARS-CoV-2 virions. Two types of aptamers—77-mer (K1) and 51-mer (M40)—were used in the study, and eight virus variants, including the wild-type, were tested. The M40 aptamer was shown not to cross-react with influenza viruses, while K1 produced false-positive signals. Therefore, FO assay with the M40 aptamer can serve as a rapid, simple, and cost-effective diagnostic tool for epidemiological surveillance of several SARS-CoV-2 variants [48].

An attempt was made to create a FP system for the direct quantitative detection of influenza A viruses using the RHA0385 DNA aptamer (with a BDPFL fluorescent label at the 5’ end). Inactivated influenza A viruses of the H1N1 (A/NewCaledonia/20/1999), H3N2 (human A/Aichi/2/1968 and avian A/duck/Moscow/4524/2011), and H7N1 (A/chicken/Rostock/45/1934) subtypes were tested as analytes. Despite the high affinity of aptamers for viruses, direct use of FP assay for their detection in a clinically relevant concentration range (10^2^–10^5^ VP/mL) proved impossible. The minimum virus concentration at which a significant increase in polarization was observed was at least 10^7^ VP/mL, which does not exceed the threshold values typical for nasopharyngeal swabs from patients (typically 10^4^–10^6^ VP/mL). The primary reason for the low sensitivity is likely related to the need to use high virus concentrations to induce a measurable change in polarization. However, the developed FP method demonstrated high efficiency in screening and ranking aptamers during their development, which is critical for selecting the best aptamers before their integration into more complex sensing platforms (e.g., SERS or electrochemical) [95]. Thus, although FP assay is often associated with DNA detection, it is a powerful and versatile tool for working with whole bacterial cells.

*Tumor Cell Detection*

In addition to infectious agents, FP analysis can be used to characterize host cells, particularly in cancer diagnostics. A promising approach is the use of methylene blue in combination with fluorescence polarization. The method is based on the ability of methylene blue to accumulate in the mitochondria of cancer cells, whose membrane potential is significantly higher than that of healthy cells. When irradiated with polarized light, the mitochondria-bound dye emits a polarized light. The mitochondrial environment is more viscous, and the dye interacts with proteins and lipids, slowing its rotation and producing a high polarization signal that correlates with the degree of malignancy. Clinical studies have confirmed the effectiveness of the method for various oncological diseases:

- Breast cancer: The FP value with methylene blue is significantly higher in malignant cells (*p* < 0.0001) and correlates with the degree of tumor differentiation, allowing cell differentiation even at minimal dye concentrations (0.01–0.05 mg/mL) [49].
- Thyroid cancer: The method helps resolve “cytologically indeterminate” cases during fine-needle aspiration, reducing the number of unnecessary surgeries [96].
- Brain cancer: Tumor cells exhibit significantly higher FP values compared to healthy astrocytes [97].
- Skin cancer: This method allows for precise delineation of the boundaries of melanoma and other non-melanoma cancers, which is critical for surgical excision [98,99].

The clinical potential of this approach is significant: it provides a quantitative (rather than subjective) assessment, reducing reliance on pathologist experience, enables real-time analysis directly in the operating room, uses an FDA-approved dye, does not affect cell viability, and can be adapted for standard confocal microscopy. Thus, methylene blue-based FP diagnostics represents a step toward personalized oncology, where treatment is prescribed based on precise molecular data, opening the door to earlier detection and successful therapy.

### 3.4. FP Assay for Detection of Antibiotic Resistance Genes

FP assay is particularly valuable for rapidly identifying genes responsible for antibiotic resistance. This directly impacts the selection of effective therapy. Highly specific FP tests have been shown to detect genes associated with drug resistance in dangerous pathogens such as Staphylococcus aureus (MRSA) and Mycobacterium tuberculosis. This allows not only detection but also precise quantification of resistance gene expression, which is important for treatment monitoring.

The One Label Extension (OLE) genotyping method, based on FP, has been adapted for Mycobacterium tuberculosis. It successfully detects mutations in six genes (e.g., rpoB, katG, inhA) responsible for resistance to rifampicin and isoniazid. An analysis of 121 clinical samples showed that the OLE test exhibits a sensitivity of 74.1% and a specificity of 98.9% for multidrug-resistant tuberculosis [100].

The main challenge lies in the need to develop highly specific DNA probes. Furthermore, the accuracy of the method can be affected by the so-called matrix effect of biological fluids, and pre-amplification (PCR) of target sequences is often required to achieve the desired sensitivity.

Thus, the FP method represents a rapidly developing alternative to traditional real-time PCR. Its ability to directly and rapidly detect genetic markers of infections and antibiotic resistance opens up broad prospects for clinical diagnostics.

## 4. New Technologies and Recognition Elements in Fluorescence Polarization Assays

Fluorescence polarization (FP) is a well-established homogeneous technique that relies on the change in rotational correlation time of a fluorophore upon binding to a larger partner. Although classical FP immunoassays—which use competitive binding and labelled antibodies—remain widely used [7,13], recent years have seen a marked shift towards novel recognition elements and miniaturized, incubation-free formats. These innovations promise to simplify workflows, reduce costs, and enable point-of-care (POC) applications. However, a critical examination reveals that significant challenges still separate proof-of-concept from routine clinical use.

The introduction of aptamers—short, chemically synthesized oligonucleotides—offers several advantages over antibodies: lower batch-to-batch variability, easier and more reproducible fluorescent labelling, and the possibility of renewable, animal-free production. Moreover, aptamers can be selected against toxic or non-immunogenic targets, expanding the scope of FP analysis.

A representative example is the work of Zhang et al. [80], who developed a nanostructured aptasensor for lactoferrin, a biomarker for dry eye syndrome. The system uses new carbon materials: carbon dots (CDs) conjugated to a lactoferrin-binding aptamer, which reportedly yields a 21.2% higher FP signal compared to unconjugated CDs. Further amplification (1.81-fold) is achieved by adding graphene oxide nanosheets (GONS), which bind to the CD-aptamer complex through non-covalent interactions. The sensor shows a linear response in the range of 0.66–3.32 mg/mL in model tear fluid and good selectivity over other proteins. While conceptually attractive, this approach has several caveats. The enhanced FP signal upon GONS addition may arise from non-specific aggregation or light scattering rather than true rotational slowing; control experiments (e.g., dynamic light scattering, lifetime measurements) are needed to confirm the mechanism. Additionally, the SELEX process for aptamer selection is not always successful, and aptamer affinity and specificity can be highly sensitive to buffer composition, temperature, and ionic strength—factors often overlooked in proof-of-concept studies. Long-term stability in real clinical samples (with variable protein and salt content) has yet to be rigorously demonstrated. Thus, although aptamer-based FP assays hold considerable promise, their translation to routine diagnostics will require careful characterization of matrix effects and shelf-life.

Beyond end-point diagnostics, FP enables real-time monitoring of biomolecular interactions. The FLAMBE (Fluorescence Polarization Ligand Assay for Monitoring BAX Early Activation) assay, developed by Gelles et al. [28], exemplifies this application. BAX is a key pro-apoptotic protein that, upon activation, undergoes conformational changes, oligomers, and forms pores in the mitochondrial membrane. Traditional methods measure end-points such as cytochrome c release or pore formation, missing the early kinetic events. FLAMBE tracks the displacement of a fluorescently labelled peptide from BAX in real time, providing high-temporal-resolution data on activation kinetics. It works in a homogeneous 96-well format, with both BH3-dependent and BH3-independent activators, and requires no specialized instrumentation. Although the assay fills a genuine methodological gap, its limitations should not be ignored. The fluorescent probe itself may subtly alter BAX conformation or binding kinetics—a common artefact in displacement assays. Moreover, the assay reports on probe release rather than direct activation, so orthogonal validation (e.g., by NMR or surface plasmon resonance) is advisable to confirm physiological relevance. The cost of labelled peptides and recombinant proteins can also be significant, and careful optimization of probe and buffer conditions is essential to avoid non-specific displacement. Nevertheless, FLAMBE is a valuable addition to the apoptosis research toolkit and can be integrated into high-throughput screening platforms for novel BAX modulators.

The inherent advantages of FP assay—homogeneous “mix-and-measure” format, rapid readout, and automation—have driven efforts to adapt it for PoC use. Microchip and microfluidic systems have been developed for therapeutic drug monitoring, including assays for theophylline [101] and tobramycin [10]. These devices achieve analysis times under 65 s, use sample volumes as low as 1 µL of whole blood, and integrate plasma separation, eliminating centrifugation steps. Correlation with reference methods (CEDIA, PETINIA) is excellent (R^2^ > 0.98).

However, practical challenges remain. Microfluidic channels are prone to clogging, especially with whole blood, and the reported coefficients of variation (~20% for tobramycin) are higher than those of clinical laboratory analyzers. Reagent stability in dried or stored form is seldom demonstrated, and calibration without onboard standards raises concerns about accuracy across different operators and lots.

On the more sophisticated end, a portable FP image analyzer that measures up to 96 samples at 1 nL per chamber was developed [102]. While technologically impressive, the 1-nL volume demands extremely precise liquid handling and optics, and the instrument’s footprint (65 × 35 × 15 cm) is hardly handheld. Its high throughput may be overkill for most PoC applications, while its complexity could be a barrier in resource-limited settings.

The development of smartphone-compatible FP detectors, such as the system for prostaglandin E2 with a detection limit of 1.57 ng/mL [103], has attracted considerable attention. By using 3D-printed holders, built-in cameras, and custom applications, these systems significantly reduce component costs and capitalize on the widespread availability of smartphones.

Nonetheless, careful scrutiny reveals several technical bottlenecks. The dynamic range and sensitivity of smartphone cameras are inferior to those of photomultiplier tubes, which limits both the lower detection limit and the usable concentration range. Ambient light and temperature fluctuations seriously affect FP readings; most prototypes require a dark enclosure and temperature control, undermining the “simple” label. Moreover, the software algorithms for polarization calculation must correct for pixel-to-pixel variations and lens aberrations—complexities rarely discussed in the literature. Therefore, while smartphone-based FP is a promising research direction, it is not yet a drop-in replacement for dedicated laboratory instruments, and its clinical adoption will depend on solving these engineering challenges.

## 5. Concluding Remarks and Future Perspectives

The reviewed literature demonstrates that fluorescence polarization (FP) technology is evolving along multiple fronts: novel biorecognition elements (aptamers, nanobodies), kinetic assays (FLAMBE), and compact hardware (microchips, portable analyzers, smartphone readers). Each innovation brings genuine benefits—speed, miniaturization, and new application domains. However, the prevailing narrative of “cost-effective, sensitive, and simple” often glosses over the practical obstacles that separate laboratory prototypes from rugged, field-ready devices. A critical assessment of the current state reveals that while FP has seen substantial technological progress, its translation into clinical practice has been slower than initially anticipated, primarily due to well-documented physicochemical constraints that are frequently underreported in proof-of-concept studies [104,105].

### 5.1. Intrinsic Limitations of Fluorescence Polarization

The fundamental theory governing FP, as described by the Perrin equation, imposes several inherent constraints that must be carefully considered during method development for target analytes [106]. First, classical FP immunoassays rely on a fluorescently labelled analogue of the target analyte that is substantially smaller than its binding partner. This limits applicability to analytes that can be chemically modified without compromising binding affinity or specificity, a challenge particularly acute for small molecules where the label may sterically hinder epitope recognition [16].

Second, biological matrices such as serum, plasma, and urine contain endogenous fluorophores (e.g., NADH, flavins, bilirubin) and scattering components that interfere with FP measurements. These background signals reduce the dynamic range, distort polarization values, and may obscure small binding-induced changes [107]. High protein concentrations also increase viscosity, artificially elevating polarization according to the Perrin relationship. Recent studies have characterized these matrix effects, demonstrating that even after standard protein precipitation or dilution, residual interference can significantly bias results for low-abundance biomarkers [35].

Third, the temperature sensitivity governed by the Perrin equation requires rigorous control, as even minor fluctuations (±0.5 °C) affect viscosity, fluorophore lifetime, and rotational correlation time, leading to reproducibility issues across different laboratories and instruments. Additionally, hydrophobic dyes may bind nonspecifically to abundant matrix proteins, generating false positive polarization signals, while the fluorescent label itself can alter the binding characteristics of the tracer, affecting apparent affinity [13,22]. Low affinity interactions present particular challenges, as they require high concentrations of unlabeled protein to achieve measurable competition, increasing solution viscosity and potentially distorting the true binding curve. Finally, for large analytes (>100 kDa), the relative change in molecular volume upon antibody binding is small, resulting in minimal polarization shifts and reduced assay sensitivity [24].

### 5.2. Mitigation Strategies and Current Validation Status

These constraints can be partially mitigated by careful assay design: (i) using smaller recognition elements (nanobodies, affibodies, aptamers) to increase the relative molecular weight change upon binding [108]; (ii) employing signal amplification strategies (nanoparticles, enzymatic amplification) to enhance sensitivity [109]; (iii) selecting fluorophores with long lifetimes (e.g., lanthanide chelates) to overcome background fluorescence; (iv) incorporating sample pre-treatment to remove interfering components [107]; and (v) rigorous temperature control and calibration using internal standards [15].

### 5.3. Priority Directions for Future Research

For the field to advance toward clinical implementation, future research should prioritize the following directions:

First, comparison of novel recognition elements (aptamers, nanobodies) with gold-standard antibodies in terms of stability, specificity, and matrix tolerance, using well-defined reference materials. Recent studies have shown that aptamer-based FP assays can achieve comparable sensitivity to antibodies while offering superior stability under ambient conditions, yet head-to-head comparisons using identical tracer designs are scarce [70]. The CRISPR/Cas system represents an emerging opportunity for FP-based detection, as Cas proteins can be engineered to recognize specific nucleic acid sequences with high specificity, potentially enabling FP readout for genetic biomarkers without the need for antibodies [110]. However, the integration of CRISPR/Cas with FP detection remains at an early proof-of-concept stage, and its compatibility with complex clinical samples requires thorough investigation.

Second, adoption of standardized protocols and cross-laboratory validation to ensure reproducibility. The lack of standardized reference materials and calibration procedures has been identified as a major impediment to the translation of FP assays from research to clinical settings [22]. The establishment of proficiency testing programs and certified reference materials, similar to those developed for other diagnostic technologies, would significantly enhance the credibility and comparability of FP-based methods [23].

Third, focus on the “last mile” engineering problems—sample pre-treatment, reagent integration, calibration, and user-interface design—rather than solely on sensitivity enhancements. Microfluidic integration offers a promising pathway to address these challenges, enabling automated sample preparation, reagent mixing, and FP detection in a single device. Recent advances in lab-on-a-chip technology have demonstrated the feasibility of integrating FP detection with on-chip sample processing, but issues related to long-term reagent stability, fluidic control, and manufacturability remain unresolved. Similarly, smartphone-based FP readers have been developed, offering the potential for truly portable and affordable diagnostics, yet current prototypes suffer from limited sensitivity compared to benchtop instruments and require careful optical alignment that may be challenging for untrained users.

### 5.4. Competitive Landscape and Strategic Positioning

Moreover, a balanced view should consider that FP is not a universal solution; it competes with electrochemical sensors, lateral flow assays, and other optical techniques, each with its own trade-offs. Electrochemical sensors offer superior sensitivity for certain analytes and are less affected by optical interferences but require more complex electrode fabrication and may suffer from biofouling. Lateral flow assays provide simplicity and low cost but are primarily qualitative or semi-quantitative. FP’s unique advantage lies in its homogeneous format, rapid readout, and capacity for quantitative measurement without extensive washing steps, making it particularly suitable for applications requiring high throughput and minimal user intervention. Its success in point-of-care settings will depend on matching the technology to the specific clinical need, the available infrastructure, and the required performance metrics. For instance, FP may be particularly advantageous in resource-limited settings for monitoring therapeutic drug levels, where quantitative results are essential and sample matrices are relatively well-characterized.

### 5.5. Concluding Assessment

In conclusion, fluorescence polarization technology has demonstrated remarkable versatility, spanning from well-established immunoassays for small-molecule drugs and hormones to emerging biosensing platforms for whole pathogens and cancer cells. The classical FPIA format for low-molecular-weight analytes is mature, supported by decades of validation, and already integrated into routine clinical laboratory workflows. In contrast, the newer applications—aptamer-based viral detection, DNA-dependent bacterial assays, and methylene blue-based tumor diagnostics—remain at the proof-of-concept or early clinical research stage. They face substantial hurdles, including matrix interference, temperature sensitivity, lack of standardized protocols, and limited cross-laboratory reproducibility, all of which must be systematically addressed before regulatory approval and widespread adoption can be considered.

The future of FP in point-of-care settings will depend less on further sensitivity enhancements and more on solving practical engineering problems—sample preparation, reagent integration, calibration, and user-friendly design—as well as on rigorous validation in real-world clinical cohorts. FP is not a universal panacea, but it occupies a valuable niche where homogeneous, rapid, and quantitative measurements are needed. With realistic expectations, sustained investment in standardization, and transparent reporting of both strengths and limitations, FP-based methods can become a reliable addition to the diagnostic toolbox, particularly for settings where infrastructure is limited and speed is critical. However, the path from laboratory innovation to clinical routine is long, and premature claims of transformative impact should be avoided.

## Figures and Tables

**Table 1:** Comparative characteristics of fluorophores for fluorescence polarization (FP).

Fluorophore	λex/λem, nm	Quantum Yield (QY)	Life Time τ, ns	Stokes Shift, nm	Sensitivity to pH	Propensity for Non-Specific Binding	Matrix Autofluorescence	Notes/Recommendations	Ref
FITC (fluorescein 5-isothiocyanate)	495/520	~0.9 (in an alkaline medium)	~4.0	~25	Strong (optimum pH 7.5–9.0)	High (binds to serum albumins)	High (serum bilirubin)	It is pH-sensitive and produces high background in plasma. Suitable for buffer systems or diluted samples.	[23,24,25]
HiLyte Fluor 647	650/665	~0.3	~1.0	~15	Weak	Moderate	Low (far red)	Long-wavelength dye; reduces autofluorescence. Suitable for serum.	[17,26]
Cy5 (cyanine)	650/670	~0.28	~1.0	~20	Weak	Moderate	Low	An analogue of HiLyte, but less photostable.	[27]
Alexa Fluor 488	495/519	~0.92	~4.1	~24	Weak (stable in the pH range of 4–10)	Low	High (like that of FITC)	More photostable than FITC and less sensitive to pH. Better suited for biological fluids.	[28,29]
Alexa Fluor 647	650/665	~0.33	~1.0	~15	Weak	Low	Low	One of the best dyes for FP in complex matrices. High photostability and low non-specific binding.	[30]
TAMRA (tetramethylrhodamine)	546/575	~0.6	~2.5	~29	Weak (stable in the pH range of 5–9)	Moderate	Moderate	Used in competitive FPIA for small molecules. Lower background than FITC.	[31]
Lanthanide chelates (Eu^3+^, Tb^3+^)	330/615	~0.5–0.7	100–1000	~250–300	Stable	Low	Extremely Low (due to temporal resolution)	The exceptionally long lifetime enables the use of time-resolved FP, completely eliminating autofluorescence. They are ideal for serum and plasma. However, they are expensive and difficult to synthesize.	[32]
Quantum dots (QDs)	350–550/500–650 (depending on size)	~0.3–0.8	10–100	>100	It depends on the surface.	It can be high (due to surface ligands).	Low (IR shift)	They enable multiplexing (different colors). A long lifetime provides signal amplification. Issues with aggregation and biocompatibility.	[27]

**Table 2:** Competitive FP assay types.

Competitive FP Assay	Recognition Element (Macromolecular Binding Partner)	Typical Analyte
FPIA (competitive immunoassay)	Antibody	Drugs, hormones, toxins
Competitive receptor-binding FP assay	Receptor or protein	Ligands, hormones
Competitive protein–ligand FP assay	Target protein	Drug candidates
Competitive aptamer FP assay	Aptamer	Small molecules, proteins
Competitive nucleic acid FP assay	DNA/RNA probe	Oligonucleotides
Competitive enzyme FP assay	Product-binding antibody or receptor	Enzyme reaction products

**Table 4:** Comparative performance of fluorescence polarization (FP) with established diagnostic platforms, based on representative studies. Ratings (++, +, −) reflect typical performance for the most common applications; actual performance varies with analyte and matrix.

Criterion	Fluorescence Polarization (FP)	ELISA	PCR	CLIA	Lateral Flow (LFA)
**Sensitivity (relative)**	++ (µM–nM for small molecules; nM for proteins with nanobodies)	+++ µM–nM	++++ (fM–aM with target amplification)	++++ (pM–fM with chemiluminescence)	+ (nM–µM visual; pM–nM with reader)
**Specificity**	++ (affinity-dependent)	+++ (matched antibody pairs)	++++ (sequence-specific primers)	+++ (antibody quality)	++ (cross-reactivity possible)
**Sample preparation**	++ (dilution, optional filtration)	+ (variable; washing steps, sometimes extraction)	+ (extensive: extraction, purification, RT)	+ (similar to ELISA)	+++ (direct application)
**Time to result**	+++ (5–30 min for most assays)	+ (2–5 h)	++ (1–3 h; qPCR 30–60 min)	+ (1–4 h)	++++ (5–20 min)
**Instrument cost**	++ (moderate; LED-based polarimeter)	++ (plate reader)	− (high: thermocycler, qPCR system)	− (high: luminometer)	++++ (very low; no reader for qualitative)
**Throughput**	+++ (automated clinical analyzers)	++ (automated ELISA handlers)	++ (high-throughput qPCR)	+++ (fully automated robotic)	+ (manual cassette)
**Clinical maturity (FDA-cleared assays)**	+++ (multiple TDM drugs, cortisol, T4)	+++ (many protein/serology assays)	+++ (many nucleic acid tests)	+++ (routine in large labs)	+++ (pregnancy, rapid antigen tests)
**Key limitation**	Matrix interference (viscosity, autofluorescence)	Washing steps, long incubation	Expensive, requires trained staff	High cost, complex instrumentation	Semi-quantitative, lower sensitivity
**Representative reference**	[7,50]	[51,52]	[53]	[54]	[55]

“++++”—excellent, “+++”—nice, “++”—good, “+” average and “−“—bad result.

**Table 5:** Strategies of signal amplification for FP analysis.

Strategy	Principle	Example Analyte (Matrix)	Achieved LOD	Amplification Factor	Assay Time	Major Limitation	Reference
**Gold nanoparticles (AuNPs)**	AuNPs bind to tracer, increase hydrodynamic volume	HIV-1 DNA (buffer)	73 pM	~10-fold vs. no AuNP	60 min	Light scattering; aggregation in serum	[46]
**Polystyrene beads**	Beads (~200 nm) attached to probe	Thrombin (buffer)	0.2 nM	~50-fold	30 min	Non-specific adsorption	[58]
**Isothermal strand-displacement (SDA)**	Exponential generation of labelled duplexes	Salmonella DNA (buffer)	10 fM	~10^6^-fold	90 min	Enzyme inhibitors; temperature control	[59]
**CRISPR/Cas12a**	Cas12a collateral cleavage of labelled ssDNA	SARS-CoV-2 RNA (buffer)	100 fM	~10^4^-fold	45 min	Only tested in buffer; not on clinical samples	[60]
**Protein aggregation**	Incorporation of labelled probe into aggregates	miRNA (buffer)	0.5 nM	~20-fold	20 min	Poor reproducibility in serum	[61]
**Target recycling (exonuclease)**	Exonuclease degrades bound probe, releases target	HIV-1 DNA (buffer)	38.6 pM	~100-fold	50 min	Requires clean samples; enzyme cost	[62]

**Table 6:** Clinical applications and perspective research of FP assays for disease diagnosis and biomarkers quantified.

Disease	Biomarker (Target)	FP Format	Clinical Purpose	Analytical Merits	Reference Methods	Validation Stage	Key References
Brucellosis	*Brucella* antibody	Direct binding FP assay using fluorescently labeled antigen	Serodiagnostics	Se 94.5% and Sp 100.0%	ELISA	Clinically validated	[66]
	*Brucella* antibody	Direct binding FP assay using fluorescently labeled antigen	Serodiagnostics	Se 96.8 and Sp 96.4%	ELISA	Limited cohort	[68]
COVID 19	SARS-CoV-2 spike protein	Direct binding FP with labeled nanobody or aptamer	Point-of-care COVID-19 antigen detection	Se ~80%, Sp ~80% LOD ~0.5 nM in buffer; >5 nM in saliva	RT-PCR	Clinically adopted	[69,70]
Acquired Immune Deficiency Syndrome	HIV-1 p24 antigen	Competitive FP assay (tracer—labeled p24)	Early HIV diagnosis in resource-limited settings	Recovery rates from 104.88% to 115.00%	-	Research	[46]
Sepsis	C-reactive protein (CRP)	Non-competitive FP using labeled Fab fragments or nanobodies	Early detection of systemic inflammation	CV < 8%, correlation with ELISA r = 0.92	ELISA/turbidimetry	Limited cohort	[17,71]
Prostate cancer	Prostate-specific antigen (PSA)	Direct binding FP with fluorescent aptamer	Prostate cancer screening and monitoring	LOD 0.01 nM; cross-reactivity with KLK2 not reported	Chemiluminescent ELISA	Research	[72]
Breast cancer	HER2/neu extracellular domain	Direct binding using Affibody	Breast cancer therapy monitoring	LOD 2.1 × 10^9^ EV/mL in human plasma	Colorimetric assay	Research	[73]
Cushing’s syndrome	Cortisol (serum, saliva)	Competitive FPIA (labeled cortisol)	Diagnosis	Se 95%, Sp 97%	LC-MS/MS	Clinically adopted	[44,74]
Thyroid disorders	Thyroxine (T4), T3	Competitive FPIA	Thyroid function testing	CV < 5%, working range 5–300 nM	Chemiluminescence	Clinically adopted	[75]
Cancer disorders	MMP-9 protease activity	FP peptide cleavage assay—decrease in polarization upon cleavage	Cancer metastasis	ND	-	Research	[76]
Coagulation disorders	Thrombin	Aptamer biosensor	Protease activity	Detection range of 0.6–100 nM, LOD 0.20 nM	-	Research	[58]
Rheumatoid disease	Lysozyme	Peptidoglycan	Protease activity		-	Research	[77,78]
	Lysozyme	Oligosaccharide	Protease activity	LOD 0.3 µM	-	Research	[79]
Dry eye	Lactoferrin	FP aptamer analysis	Eye diagnostics	LOD 17 nM	-	Research	[80]
Cancer disorders	Transferrin	Nanobody	Buffer	Detection range apo-Tf 0.125–0.750 µM holo-Tf 0.125–12.50 µM	ELISA	Research	[24]
Immunological disorders	IgG, IgA	Nanobody	Plasma	Detection range IgA 0.23–0.80 µM IgG 0.50–1.76 µM	ELISA	Research	[25]

Se—sensitivity and Sp—specificity, research—buffer/serum only, <50 samples; limited cohort—>50 clinical samples, but no independent validation. Clinically validated—prospective study of >100 samples, comparison with the gold standard; clinically adopted—regulatory approval (FDA, CE, NMPA) and a commercial kit.

## Data Availability

No new data were created or analyzed in this study. Data sharing is not applicable to this article. All data cited in this review are available from the original publications referenced herein.

## Abbreviations

The following abbreviations are used in this manuscript:

CD	Carbon dot
CLIA	Clinical Laboratory Improvement Amendments
CRP	C-reactive protein
DNA	Deoxyribonucleic acid
FP	Fluorescence polarization
FPA	Fluorescence polarization assay-any assay that use FP
FPIA	Fluorescence polarization immunoassay
FITC	Fluorescein isothiocyanate
HIV	human immunodeficiency virus
HBV	Hepatitis B virus
hLF	Human lactoferrin
Apo-TF	Apo-transferrin
Holo-TF	Holo-transferrin
PSA	Prostate specific antigen
PoC	Point of care
RNA	Ribonucleic acid
Se	Sensitivity
Sp	Specificity
LOD	Limit of Detection
